# On the Origin of DNA Genomes: Evolution of the Division of Labor between Template and Catalyst in Model Replicator Systems

**DOI:** 10.1371/journal.pcbi.1002024

**Published:** 2011-03-24

**Authors:** Nobuto Takeuchi, Paulien Hogeweg, Eugene V. Koonin

**Affiliations:** 1 National Center for Biotechnology Information, National Library of Medicine, National Institutes of Health, Bethesda, Maryland, United States of America; 2 Theoretical Biology and Bioinformatics Group, Utrecht University, Utrecht, The Netherlands; University of Texas at Austin, United States of America

## Abstract

The division of labor between template and catalyst is a fundamental property of all living systems: DNA stores genetic information whereas proteins function as catalysts. The RNA world hypothesis, however, posits that, at the earlier stages of evolution, RNA acted as both template and catalyst. Why would such division of labor evolve in the RNA world? We investigated the evolution of DNA-like molecules, i.e. molecules that can function only as template, in minimal computational models of RNA replicator systems. In the models, RNA can function as both template-directed polymerase and template, whereas DNA can function only as template. Two classes of models were explored. In the surface models, replicators are attached to surfaces with finite diffusion. In the compartment models, replicators are compartmentalized by vesicle-like boundaries. Both models displayed the evolution of DNA and the ensuing division of labor between templates and catalysts. In the surface model, DNA provides the advantage of greater resistance against parasitic templates. However, this advantage is at least partially offset by the disadvantage of slower multiplication due to the increased complexity of the replication cycle. In the compartment model, DNA can significantly delay the intra-compartment evolution of RNA towards catalytic deterioration. These results are explained in terms of the trade-off between template and catalyst that is inherent in RNA-only replication cycles: DNA releases RNA from this trade-off by making it unnecessary for RNA to serve as template and so rendering the system more resistant against evolving parasitism. Our analysis of these simple models suggests that the lack of catalytic activity in DNA by itself can generate a sufficient selective advantage for RNA replicator systems to produce DNA. Given the widespread notion that DNA evolved owing to its superior chemical properties as a template, this study offers a novel insight into the evolutionary origin of DNA.

## Author Summary

At the core of all biological systems lies the division of labor between the storage of genetic information and its phenotypic implementation, in other words, the functional differentiation between templates (DNA) and catalysts (proteins). This fundamental property of life is believed to have been absent at the earliest stages of evolution. The RNA world hypothesis, the most realistic current scenario for the origin of life, posits that, in primordial replicating systems, RNA functioned both as template and as catalyst. How would such division of labor emerge through Darwinian evolution? We investigated the evolution of DNA-like molecules in minimal computational models of RNA replicator systems. Two models were considered: one where molecules are adsorbed on surfaces and another one where molecules are compartmentalized by dividing cellular boundaries. Both models exhibit the evolution of DNA and the ensuing division of labor, revealing the simple governing principle of these processes: DNA releases RNA from the trade-off between template and catalyst that is inevitable in the RNA world and thereby enhances the system's resistance against parasitic templates. Hence, this study offers a novel insight into the evolutionary origin of the division of labor between templates and catalysts in the RNA world.

## Introduction

At the core of all biological systems lies the division of labor between the storage of genetic information and the manifestation of genetic information, i.e. the functional differentiation between DNA, which is the information storage medium (template), and RNA and proteins, which are responsible for different aspects of operation (catalyst). This fundamental property of life, however, is believed to have been absent at the earliest stages of evolution. The RNA world hypothesis, which is currently considered to be the most, if not the only, realistic scenario for the origin of life, posits that, in the first, primitive replicating systems, both the storage of genetic information, and chemical catalysis were embodied in a single type of molecules, namely, RNA [1]–[5]. According to this hypothesis, DNA and proteins evolved later as specialized components dedicated to information storage and chemical catalysis, respectively, thereby achieving the division of labor between templates and catalysts. The emergence of this division marks a pivotal event among the major transitions of evolution [6]. The RNA world hypothesis has stimulated extensive studies of reactions catalyzed by natural and synthetic ribozymes which revealed a remarkable, previously unsuspected diversity of catalytic activities of RNA [7]–[10]. The catalytic versatility of ribozymes cannot validate the RNA World hypothesis but clearly is compatible with this scenario.

What selective advantage could there be for the evolution of DNA and proteins in the RNA world? Proteins are obviously superior to RNA as chemical catalysts because of their greater repertoire of chemical moieties and structural flexibility. Conversely, proteins are vastly inferior to RNA for the storage of genetic information because of the absence of mechanisms for template-directed replication. These properties of proteins are compatible with the view that proteins evolved as entities specialized in chemical catalysis owing to their superiority to RNA in that capacity.

The case of DNA appears less straightforward. On the one hand, it remains somewhat unclear what would be the principal driving forces behind the evolution of DNA in the RNA world. DNA is generally a less reactive molecule than RNA thanks to the absence of the 2′-hydroxyl at its sugar moiety. In particular, DNA is markedly more resistant to hydrolysis than RNA [11], especially in the presence of metal ions [12], which would certainly be important components of the RNA world given the ion requirement for most of the catalytic activities of ribozymes. Hence, it is often suggested that DNA has an advantage over RNA as a medium of genetic information storage [13]. However, Forterre recently argued that the greater stability of DNA could not account for the origin of DNA because the advantage of employing DNA for information storage lies in the possibility of evolving a longer genome, which in itself would not provide any immediate selective advantage to the systems that included DNA [14]. The possibility to correct C to U misincorporation is often considered to be another advantage of DNA [13]. However, such correction requires specialized catalytic machinery and so, again, could not provide a short-term advantage within the context of the RNA world. Forterre also proposed an alternative scenario, in which viruses evolved DNA genomes under the pressure to evade defense systems of the hosts [14]. This hypothesis is predicated on the existence of complex RNA cells encoding, among other functions, the defense systems. However, RNA cells might not be a realistic stage in the evolution of life for a variety of reasons [15].

On the other hand, there is no clear experimental evidence demonstrating that DNA is inferior to RNA as a chemical catalyst [16]. DNA molecules that can catalyze various chemical reactions have been successfully produced in *in vitro* evolution experiments [10], [17]–[19]. Hence, the chemical properties of DNA do not necessarily conduce to the fact that the function of DNA is restricted to information storage.

Given these considerations, we ask: What selective advantage could there be for an RNA-based evolving system to evolve an entity that is solely dedicated to the storage of genetic information, i.e., an entity that is *functionally* equivalent to DNA?

As a first attempt to answer this question, we consider the evolution of DNA-like molecules in RNA replicator systems, the simplest form of the RNA world that can undergo Darwinian evolution. Our aim is to examine whether there could exist purely population dynamical factors, independent of specific nucleic acid chemistry, which would support selection for DNA-like molecules, i.e., molecules solely dedicated to the storage of information, in RNA replicator systems. To address this question, we construct and investigate minimal computational models of an RNA-like replicator system with a built-in possibility to evolve DNA-like molecules.

## Models

There are two types of molecules in the models developed here: “RNA-like molecules” and “DNA-like molecules” (RNA and DNA, respectively, for short). The only difference between the two types of molecules is the presence or absence of the catalytic capacity—all other possible differences are ignored for the sake of simplicity and focus. Thus, an RNA molecule can be both a template for replication and a catalyst that replicates other templates, whereas a DNA molecule can only be a template for replication (to replicate templates is the only catalytic function considered in the models). Moreover, DNA and RNA compete for a common resource (precursors) for replication (this direct competition between DNA and RNA is expected to make the models more conservative with respect to the evolution of DNA). The models do not include protein-like molecules because we intend to investigate the simplest possible scenarios under which the evolution of DNA can be considered (see the “Discussion” section for more on this point).

The two types of molecules give rise to four types of replication reactions, namely:

1. RNA-dependent RNA synthesis,
2. RNA-dependent DNA synthesis,
3. DNA-dependent RNA synthesis,
4. DNA-dependent DNA synthesis.

To focus on the population dynamical aspect of the problem, we ignore all specific details of the molecular mechanisms [20], [21] of these distinct polymerization reactions and make the following simplification (see the “Discussion” section for more on this point). Regarding the substrate specificity, a replicase is either an RNA polymerase or a DNA polymerase (Rp or Dp, respectively, for short); i.e., the same catalyst cannot produce both RNA and DNA molecules. However, the type of polymerase can be converted from one to another as a result of rare mutations (see below). Regarding the template specificity, a replicase has a potential to discriminate between RNA and DNA. However, for simplicity, it is assumed that replicases do not discriminate between different DNA templates and between different RNA templates (to take account of such discrimination would make the model too complex for the purpose of the current work). It should be noted that, although a catalyst is always RNA, the information on a catalyst can be stored either in an RNA template (which itself is the catalyst) or in a DNA template. Thus, to distinguish between the RNA-form and DNA-form of catalysts, we use superscripts as follows: Rp^RNA^ and Rp^DNA^, and Dp^RNA^ and Dp^DNA^. When it is preferred not to distinguish between these two forms, catalysts are simply referred to without superscripts.

The replication reaction is assumed to occur in two steps, namely complex formation between a template and a catalyst (replicase) and actual replication of the template:
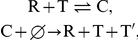
where 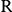 denotes a replicase; 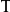 denotes a template; 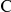 denotes a complex between 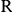 and 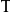, and 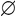 denotes resource for multiplication; 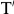 is the newly produced copy of 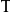, which can be either RNA or DNA depending on the type of polymerase 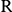 (in real replication processes, the template and the product are complementary to each other; however, for simplicity, the models ignore this, so 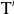 is identical to 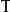 if no mutation occurs). Including the complex formation allows us to take into account the fact that replication is not an instantaneous process [22], [23]. The template specificity of a replicase is specified by the rate constant of complex formation between 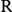 and 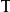. Each replicase is assigned two parameters: 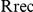 and 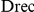 for RNA and DNA recognition, respectively. If 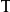 is RNA, the rate constant of complex formation is the value of 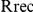 of 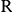; otherwise, it is the value of 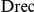 of 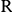. The rate constant of complex dissociation is set to 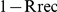 and 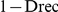, respectively. 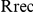 and 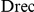 assume values between 0 and 1, ranging from the case of no complex-formation to the case of no complex-dissociation, respectively. Once a complex is formed between 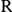 and 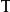, replication occurs with the rate constant 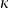. The value of 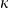 is assumed to be identical regardless of the type of a replicase and a template that form a complex.

A molecule produced by replication (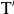) inherits the properties of the template from which it is produced (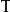), but the properties can be modified by mutation, which occurs with a certain probability during replication. There are four types of mutations that are mutually exclusive: a change in the value of 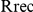 (the probability of which is 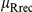), a change in the value of 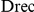 (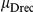), conversion of the type of a replicase (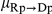 and 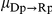), and conversion of a replicase into an inactivated form, a parasite (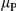) (see below). A change in 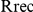 and 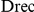 is obtained by adding a random number uniformly distributed in 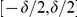 (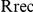 and 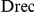 are bounded in 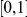; see Text S1 for details). For simplicity, we set 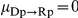 because this type of mutation is not required for the evolution of DNA molecules in the present models (setting 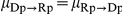 did not qualitatively change the results because the population of Rp did not go extinct; data not shown).

In addition to the replication reaction, the decay reaction that converts replicators into the resource occurs with a rate constant 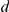: 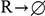 and 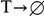. The decay of complex molecules is treated as independent decay of the constituent molecules: 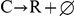 and 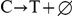.

The class of replicators customarily called “parasites” is known to play important roles for the evolutionary dynamics of RNA-like replicator systems [24]–[27]. Parasites are molecules that do not catalyze replication of other molecules but can be replicated by the catalysts, possibly at a faster rate than the catalysts themselves. Under well-mixed conditions, the parasite can bring a replicator system to extinction by (over)exploiting catalysts (e.g., see [22]). Because of this inherent instability of RNA-like replicator systems against the parasite, it is necessary to consider spatial structure in the population of replicators and the discreteness of the population, which can prevent the extinction caused by parasites [28]–[32]. Moreover, if extinction is prevented through spatial pattern formation, the parasite can contribute to the evolution of complexity in RNA-like replicator systems [24].

Given these previous studies, we introduced parasites into the models. The models assume a special class of molecules, parasites, that have no catalytic activity but have an increased rate of complex formation with catalysts by a constant factor 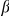; e.g., if a parasite is RNA, the complex formation rate is 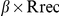, where 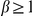 (the complex dissociation rate is unaffected and is 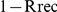).

The model replicator system specified above was implemented as a spatially extended, individual-based stochastic simulation model. Two models were constructed: one in which replicators are assumed to be confined on a surface with finite diffusion (the surface model, for short) and another in which replicators are compartmentalized by vesicle-like boundaries that are impermeable to replicators (the compartment model). In the compartment model, the size of a compartment grows (or shrinks) in proportion to the number of replicators inside the compartment, and a compartment divides when its size reaches a threshold given by a parameter 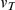. The surface model does not assume any factors other than the birth, death and diffusion of replicators and so is simpler than the compartment model. However, the compartment model has an obvious relevance to the recent experimental efforts to synthesize model “protocells” (for reviews, [33], [34]).

The two models were implemented as described previously [35] (see Text S1, for details). Briefly, the surface model was implemented in two-dimensional cellular automata (CA). One square of the CA contained at most one replicator, and empty squares were considered to represent the resource (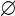); hence, the number of replicators the system could sustain was limited both locally and globally. The dynamics were run by consecutively applying an algorithm that locally simulates the reactions specified above and diffusion. Interactions occurred only between molecules that were adjacent to each other on the CA grid. Diffusion was implemented as exchange of contents between adjacent grid squares, and the rate of diffusion is given by the parameter 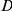. Both reactions and diffusion were prohibited to occur across CA and compartment boundaries.

To simulate the dynamics of compartment boundaries, we employed the Cellular Potts Model (CPM) [36], [37]. The CPM was implemented in two-dimensional CA. Each compartment consisted of a set of grid squares with identical states. The CPM algorithm tends to bring the size of each compartment (i.e. the number of squares that constitute a compartment) closer to its target size while minimizing the number of contacts between different compartments. The CPM was superimposed onto the surface model to generate the compartment model. The value of 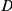 was increased so that the internal replicator system within a compartment was relatively well-mixed. The dynamics of compartment boundaries and those of replicators were coupled by setting the target size of a compartment to be proportional to the number of replicators present in the compartment with the factor of proportionality 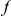 (see [38], for an experimental support of this coupling). When the size of a compartment reached the threshold (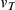), the compartment was divided along the line of the second principal component; the internal replicators were distributed between the two daughter compartments according to their location.

## Results

**Figure 1:**
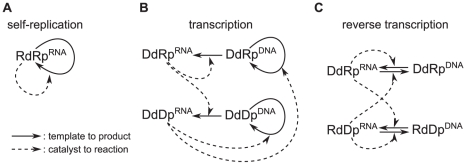
The three replicator systems made of the four types of polymerases. The notation is as follows: Rp and Dp denote RNA polymerase and DNA polymerase respectively. The superscripts to Rp and Dp denote whether the polymerase is in RNA-form (catalyst and template) or in DNA-form (template). The prefixes to Rp and Dp denote the type of templates a polymerase depends on: Rd stands for RNA-dependent, and Dd stands for DNA-dependent. Solid arrows represent the template-product relationship. Broken arrows represent the catalyst-reaction relationship. A: Self-replication system consists of an RNA replicase (RdRp). B: Transcription system consists of a transcriptase (DdRp) and a DNA replicase (DdDp). C: Reverse transcription system consists of a transcriptase (DdRp) and a reverse transcriptase (RdDp).

Before presenting the results of the simulations, let us first consider replicator systems consisting of RNA and DNA in general terms. In such a replicator system, there are four replication reactions as listed in the Models section. These four reactions provide for three types of replicator systems, which we denote the self-replication system, the transcription system and the reverse transcription system (Figure 1). The self-replication system consists only of RNA molecules that function both as the templates and as the RNA-dependent RNA polymerases (Figure 1A). This system is “primitive” in the sense that both genetic information and chemical catalysis are provided by a single type of molecules, so there is no division of labor between templates and catalysts. In contrast, the transcription system consists of both RNA and DNA and establishes a division of labor between the template and the catalyst (Figure 1B), where RNA plays the role of the catalyst whereas DNA plays the role of the template. An intermediate case is represented by the reverse transcription system (Figure 1C), which contains DNA molecules but requires RNA molecules to function both as catalyst and as template to complete the replication cycle.

By comparing the three replicator systems, we can see two effects that can be brought about by the inclusion of DNA molecules into a replication cycle. First, the systems that include DNA are more complex and thus less efficient than the self-replication system. The inclusion of DNA requires the joint action of four types of molecules to complete a replication cycle (Rp^RNA^, Rp^DNA^, Dp^RNA^ and Dp^DNA^), regardless of whether replication proceeds via the transcription cycle or via the reverse transcription cycle. Assuming that the total concentration of molecules is constant, this increase in the complexity of the replication cycle leads to a reduction in the concentration of each type of molecules and to the corresponding reduction in the rate of multiplication compared to the self-replication system.

Second, however, there is a converse effect: the division of labor between the template and the catalyst, which emerges in the transcription system, releases the system from a trade-off that exists in the self-replication system. Because replication is not an instantaneous process, a catalyst spends a part of its lifetime replicating other molecules, and during these times, the catalyst itself cannot be replicated [24]. In the self-replication system (the RNA-only cycle), catalysts also serve as templates to be replicated; therefore, the system is hampered by the trade-off between the RNA molecules spending time as templates and as catalysts (the trade-off between template and catalyst for short). This trade-off gives a substantial advantage to a parasite, which spends all of its lifetime being a template [22]. By contrast, the transcription system is free of such a trade-off: the catalysts (RNA) are produced by transcription of the DNA and so do not have to spend any time being templates in order to complete the replication cycle. In the reverse transcription system, however, the catalysts (RNA) also serve as templates in order to produce DNA via reverse transcription. Hence, the reverse transcription system does not establish the division of labor between the template and the catalyst (but, similarly to the transcription system, it suffers from the reduction in the rate of multiplication due to the increased complexity of the replication cycle).

In the following section, we use the described models to examine whether a replicator system, starting from the simple self-replication (RNA-only cycle), can evolve DNA molecules and the ensuing division of labor between the template and the catalyst.

### The surface model

#### Evolution of the transcription system in the surface model

**Figure 2:**
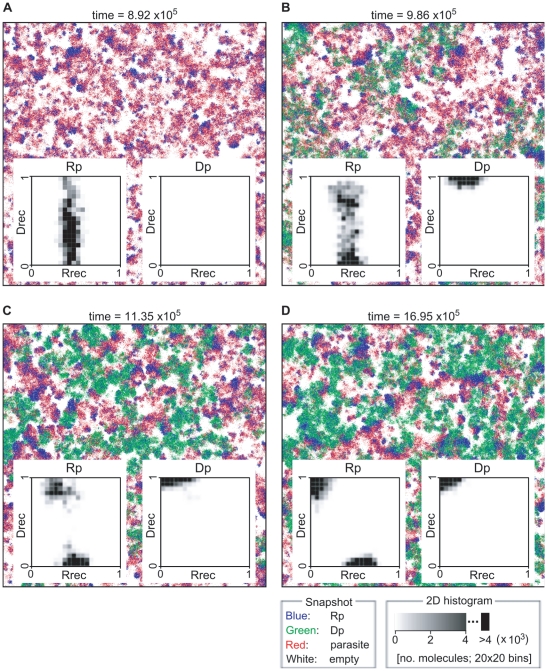
The evolution of the transcription system in the surface model. The model was initialized such that the system consisted of a population of RNA polymerase (Rp) and parasites. The simulation was first run with the mutation converting Rp into Dp disabled (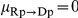). After the system reached evolutionary equilibrium (panel A), the mutation was enabled (
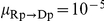
), and the resulting evolutionary dynamics are depicted in panel B to D. The larger panels depict snapshots of simulations taken at different times as indicated above panels. The color coding is indicated at the bottom of the figure. RNA and DNA are not distinguished. The timescale is scaled such that it has the same meaning as that of the ordinary differential equation model that describes the replicator dynamics with the same rate constants as in the CA model (the timescale is scaled in this manner throughout the paper). The smaller panels within the larger panels depict a two-dimensional histogram of 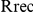 and 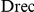. See the main text for the description for each panel. The parameters (rate constants) used in this simulation were as follows: 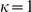 (replication); 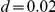 (decay); 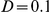 (diffusion); 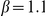 (parasite advantage); 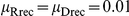 (mutation rate of 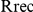 and 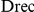); 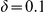 (mutation step); 
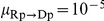
 (mutation rate from Rp to Dp); 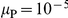 (mutation rate to parasites). The size of CA was 1024×1024 squares. The boundary had no flux.

The surface model was initialized with a homogeneous population of Rp^RNA^ with arbitrary chosen values of 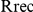 and 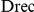 (Table 1, No. 1). A simulation was first run with the mutation from Rp to Dp disabled. In this simulation, the system contained a large number of parasites, and the spatial distribution of catalysts and parasites formed traveling wave patterns (Figure 2A). The front of a wave consists of Rp^RNA^, and it expands into an empty region as the population of Rp^RNA^ grows. The back of a wave consists of parasites, and it contracts, leaving empty regions, in the direction of wave propagation due to the extinction caused by parasites [22] (under well-mixed conditions, the system is unstable as mentioned in the “Models” section; see also Figure 9A). When the evolutionary dynamics reached equilibrium (Figure 2A), the population of Rp displayed a unimodal distribution of 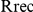 (Text S1, Note 1) and a uniform distribution of 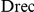 (a trivial consequence of the absence of DNA in the system). After the equilibrium was reached, the mutation from Rp to Dp was enabled. The system then displayed the following evolutionary dynamics. Given that the distribution of the 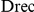 of Rp was uniform, a mutation (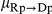) could produce Dp with various values of 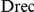. The Dp that had relatively greater values of 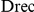 invaded the system (Figure 2B) and quickly evolved towards specialization on DNA replication, i.e., increasing the value of 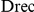 and decreasing the value of 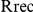 (Figure 2C). In other words, the original, dual specificity Dp that emerged as the result of the mutation of Rp evolved into a DNA replicase. Upon the invasion of Dp, the population of Rp diverged into two populations that have markedly different distributions of 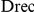 (Figure 2C), where one population (dual specificity Rp) recognized DNA templates well (high 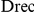 values), whereas the other population (RNA replicase) recognized almost only RNA templates (low 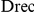 values). Subsequently, the dual specificity Rp evolved towards recognizing only DNA templates by reducing the value of 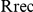, i.e., became a transcriptase (Figure 2D). The net outcome is the evolution of the system into a state in which the two types of replicator systems, namely, the self-replication system (with the RNA replicase as the only catalyst) and the transcription system (with two distinct catalysts, a transcriptase and a DNA replicase), stably coexist with one another and with parasites.

**Table 1:** Summary of the results with the surface models.

No.	Purpose of simulation	Setting of simulation	Ref.	Results
1	Standard simulation. Point of reference	Starting with self-replication system	Fig. 2 & Fig. 6B	Transcription system evolved, and it coexisted with self-replication system.
2	To observe the short-timescale dynamics of transcription system evolved in No. 1	Idealized transcription system (no mutation)	Fig. 3	Transcription system was resistant against parasites, but produced many empty regions.
3	To examine the role of parasites for the coexistence observed in No. 1	Parasites were removed in No. 1 after reaching equilibrium (no mutation)		Transcriptase (DdRp) went extinct: transcription system was destabilized.
4	To examine the role of self-replication system for the evolutionary stability of transcription system	Self-replication system was removed in No. 1 after reaching equilibrium	Fig. 4 & Fig. 6C	Transcription system regenerated self-replication system: DdRp became evolutionary unstable and diverged into RdRp & DdRp via dual-Rp.
5	To examine the role of reverse transcription activity for the evolutionary destabilization of transcription system	The same as No. 4, except that reverse transcription was completely suppressed	Text S1, Note 4	Transcription system did not regenerate self-replication system: DdRp remained evolutionarily stable.
6	To examine the role of parasites for the evolution of transcription system	The same as No. 1, except that the model excluded the predefined parasite	Fig. 5 & Fig. 6D	Transcription system evolved, enabling self-replication system to diverge into a catalytic and parasitic species.
7	To examine the effect of complex formation on the evolution of DNA	The model assumed that replication was an instantaneous process.		DNA did not evolve: complex formation is important for the evolution of DNA

#### Parasites enable the transcription system to coexist with the self-replication system

**Figure 3:**
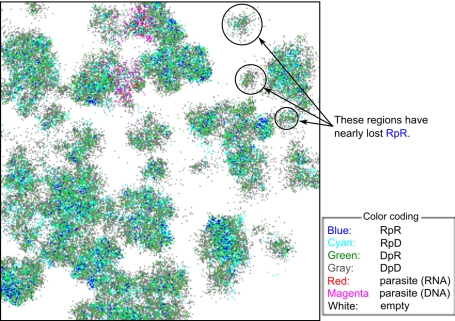
Spatial pattern generated by the transcription system in the absence of the self-replication system. The surface model was initialized such that the system consisted of the transcription system (see below for the parameter values). No mutation processes were enabled except for the mutation converting molecules into parasite (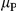). The color coding is indicated in the figure. The parameters were as follows: 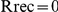 and 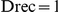 for both Rp and Dp; 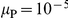; 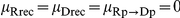; the size of CA was 512×512 squares; the other parameters were the same as in Figure 2.

Given that every replicator competes for the same resource (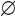), how can the two replicator systems coexist? To elucidate the mechanism of the coexistence, we ran the following simulation (Table 1, No. 2). A system was initialized with a homogeneous population of an idealized, pure transcription system (the values of 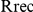 and 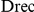 were set to 0 and 1, respectively, for both Rp and Dp). Mutations were disabled except for those converting catalysts into parasites. In this simulation, the transcription system displayed a distinct spatial pattern with numerous “clumps”, which mainly consisted of Dp^DNA^, and slowly grew, split and occasionally shrank and disappeared (Figure 3). The system contained relatively large empty regions (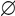) between the clumps (this was the case even when parasites were absent as becomes obvious from the inspection of the spatial distribution of parasites in Figure 3). This result indicates that the multiplication of the transcription system is inefficient, which appears to originate from the increased complexity of replication cycle through the inclusion of DNA (as described in the previous section) and from the finiteness of diffusion (see Text S1, Note 2, for details). Moreover, the transcription system contained a far smaller number of parasites compared to the self-replication system (cf. Figure 2A); if the influx of parasites through mutation was eliminated, the parasite soon went extinct under the parameters of this simulation. This indicates that the transcription system is resistant against parasites. This can be explained as follows. In the transcription system, the division of labor between templates and catalysts is established, so that the templates (Rp^DNA^ and Dp^DNA^) do not spend any part of their lifetimes replicating others, which reduces the advantage of the parasite over the catalyst (the parasite still has some advantage given that 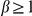) (see Text S1, Note 3, for additional discussion).

Given the above properties of the transcription system, its coexistence with the self-replication system can be rationalized as follows. First of all, the transcription system is more resistant to parasites. In particular, the parasites that exploit the self-replication system are all RNA templates, so they cannot exploit the transcription system (which replicates DNA templates). Hence, when a traveling wave consisting of Rp^RNA^ and the parasite hits a clump consisting of the transcription system, the clump can remain intact whereas the traveling wave is annihilated because the expansion of the wave front is impeded by the lack of resource (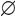) in the regions that are already occupied by the transcription system. However, the transcription system produces large empty regions in the system due to its inefficiency of multiplication. In contrast, the self-replication system can multiply faster, so the traveling waves can propagate into those empty regions before the transcription system expands into those regions. In this way, the self-replication system can thrive along with the transcription system. This mechanism of coexistence is similar to that suggested for grassland ecosystems by Tilman [39], wherein a faster reproducing species was able to thrive along with a slower reproducing species. The latter was able to outcompete the faster growing species locally due to K-selection—in the present models, this is due to differential resistance against parasites—but which always left some area in the system unoccupied due to its slower growth and the occasional local extinction.

The above explanation implies that parasites are one of the key factors behind the coexistence of the self-replication and transcription systems. To examine whether this is indeed the case, we conducted the following simulation (Table 1, No. 3). A system was initialized with populations of the idealized, pure self-replication system (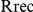 and 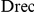 were set to 0.4 and 0, respectively) and the idealized, pure transcription system (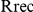 and 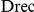 were set to 0 and 1, respectively). We compared the dynamics of the system between the cases with and without parasites, with all types of mutations disabled. In this experiment, the absence of parasites caused the extinction of the transcriptase, in support of the hypothesis that parasites are essential for the coexistence of the two replicator systems. The DNA replicase did not go extinct in the absence of parasites because Dp^RNA^ can parasitize on the self-replication system given that its 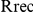 value is 0 as is the case for parasites by definition. Thus, the short-term, “ecological” stability of the transcription system in the presence of the self-replication system is mediated by the parasites that exploit the self-replication system (“ecological” pertains to the absence of mutation processes; below we discuss the long-term, “evolutionary” stability).

#### The self-replication system evolutionarily stabilizes the transcription system

**Figure 4:**
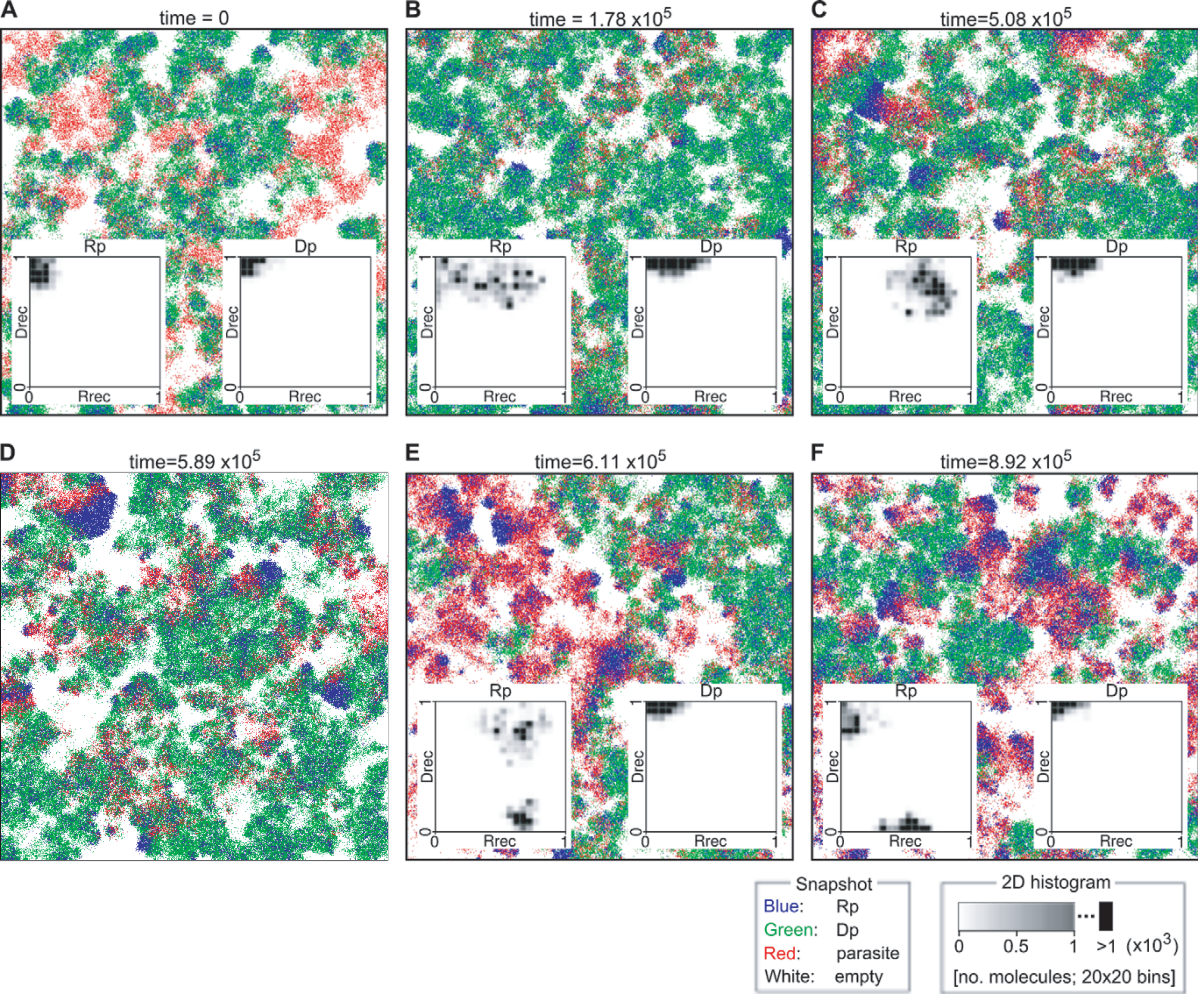
The evolutionary dynamics of the transcription system after the self-replication system was removed. After the surface model reached evolutionary equilibrium (Figure 2D), the whole population of the RNA replicase (i.e. the self-replication system) was removed from the system (Figure 4A), and the simulation was continued. The resulting evolutionary dynamics are depicted (Figure 4B–F). The figure has the same format as that of Figure 2. See the main text for the explanation of each panel. The parameters where as follows: 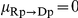; the size of CA was 512×512 squares; the other parameters were the same as in Figure 2.

Can the transcription system supersede the self-replication system? In other words, can a transcription system, assuming that it emerged, be maintained through evolution in the absence of the self-replication system? The answer turns out to be negative. To address this question, we continued the previous simulation, in which the self-replication system and the transcription system coexisted, by removing the entire population of the self-replication system (Table 1, No. 4). The result was that the transcriptase evolved towards improved recognition of RNA templates, thus “re-inventing” RNA replication (Figure 4ABC). After the transcriptase evolved into a dual specificity Rp, a subset of its population evolved to reduce its transcription activity (decreased value of 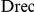), becoming an RNA replicase (Figure 4CDE). After the RNA replicase evolved, the remaining dual specificity Rp evolved into a transcriptase. Thus, the system eventually returned to the original state through re-evolving the self-replication system. To summarize the results of this simulation, the self-replication system is required for the stable evolutionary maintenance of the transcription system: when the self-replication system is eliminated, the transcription system evolves the self-replication system via the evolution of a dual specificity Rp from the transcriptase, which results in the stabilization of the transcription system.

How and why does the presence of the RNA replicase cause the evolutionary stabilization of the transcriptase? The RNA replicase has two distinct effects on the transcription system:

1. The catalysts of the transcription system (transcriptase Rp^RNA^ and DNA replicase Dp^RNA^) are directly replicated by the RNA replicase;
2. If the transcriptase evolves into a dual specificity Rp, it will replicate the RNA replicase Rp^RNA^.

To determine which of these two effects causes the evolutionary stabilization of the transcriptase, we modified the model such that interactions between the RNA replicase and the transcriptase were prohibited. The following three cases were investigated: 1) The RNA replicase does not recognize the transcriptase as a template; 2) the transcriptase does not recognize the RNA replicase as a template (both RNA and DNA given that it can evolve into a dual specificity Rp); 3) the RNA replicase and the transcriptase do not recognize each other (a combination of 1 and 2). For each of these three cases, we continued the previous simulation that had reached the evolutionary equilibrium. The results showed that the evolutionary stabilization of the transcriptase was obtained only in case 1. In this case, the modification did not qualitatively change the behavior of the system although the transcriptase went extinct after a long time due to fluctuation. In case 2, the transcriptase evolved into a dual specificity Rp, which out-competed the original RNA replicase, and which then underwent speciation into the RNA replicase and the transcriptase, re-establishing the original system as we saw before (note that the model allowed interactions between the descendants of the original transcriptase). In case 3, the transcriptase evolved into a dual specificity Rp, which went extinct after its value of 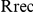 exceeded that of the RNA replicase. Therefore, the evolution of the transcriptase into a dual specificity Rp is prevented because a dual specificity Rp replicates the RNA replicase Rp^RNA^. This duality makes the dual specificity Rp selectively inferior to the transcriptase which does not waste time and resource (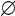) on replicating RNA replicase Rp^RNA^. (See Text S1, Note 4, for additional information.)

#### The transcription system induces the evolution of parasites

**Figure 5:**
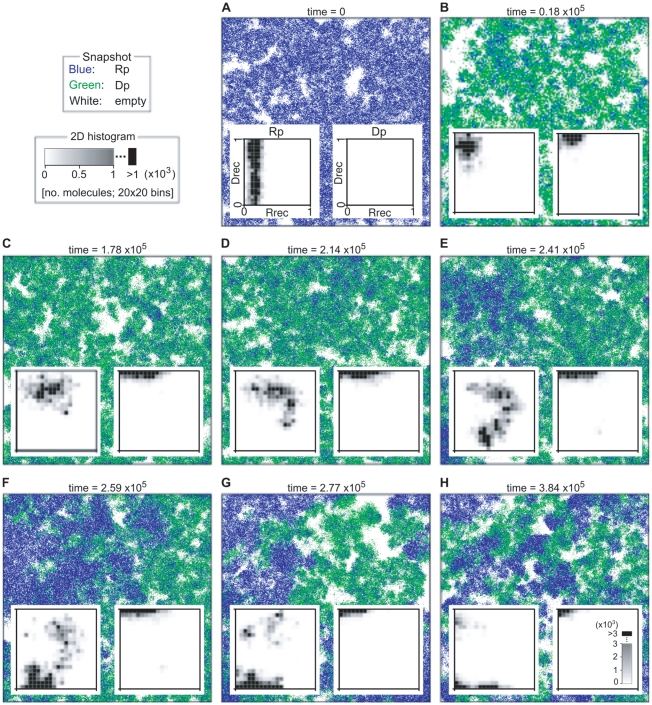
The evolutionary dynamics of the surface model without explicitly predefined parasites. The surface model was initialized with a population of Rp (no parasites were introduced in the system). The simulation was run in the same manner as in Figure 2 with the mutation converting molecules into parasites disabled (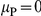). The format of the figure is the same as that of Figure 2. For the explanation of each panel, see the main text. The parameters were as follows: 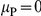 ; the size of CA is 512×512 squares; the other parameters were the same as in Figure 2.

As shown above, parasites mediate the coexistence between the self-replication system and the transcription system. Does this mean that the evolution of Dp was caused by parasites? To analyze the cause and effect relationship between the parasite and the evolution of the transcription system, the model was modified to exclude parasites that are explicitly defined as a class of non-catalytic replicators with an advantage (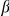) for the recognition by catalysts (Table 1, No. 6). In this model, the difference between catalysts and parasites is continuous as it is determined by the value of 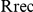 and 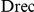. Thus, only quantitative distinction can be made between catalysts and parasites: the catalysts that recognize templates less well are “more parasitic” than those that recognize templates better. As before, the simulation was first run until it reached equilibrium with the mutation converting Rp into Dp disabled. In this simulation, the system did not develop a traveling wave pattern; instead, it exhibited continuous production and refilling of small empty spots (Figure 5A). Moreover, the RNA replication activity of the Rp (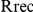) was distributed around a single peak; hence, there was no sharp boundary between catalysts and parasites. The RNA replication activity was significantly lower than that which evolved when the system contained parasites as can be seen from the comparison of the value of 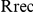 between Figure 2A and Figure 5A (Text S1, Note 5).

We then enabled the mutation converting Rp into Dp. The resulting system displayed the following evolutionary dynamics. Dp quickly invaded the system, evolving into a DNA replicase (Figure 5B). Upon the invasion of Dp, the population of Rp that had higher transcription activity (greater values of 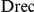) out-competed the population of Rp that had lower transcription activity (smaller values of 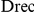) (compare Figure 5A and Figure 5B). This is in contrast to the model containing explicitly defined parasites, in which the population of Rp underwent speciation with respect to the distribution of the values of 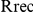 (Figure 2BC). After the evolution of the DNA replicase, the remaining population of Rp evolved towards increasing RNA replication activity (Figure 5C). After Rp increased the RNA replication activity sufficiently (i.e., it evolved into a dual specificity Rp), a subpopulation of it started to evolve towards decreasing transcription activity, becoming an RNA replicase (Figure 5DEF). After the RNA replicase evolved, the remaining dual specificity Rp evolved towards decreasing RNA replication activity, becoming a transcriptase (Figure 5FGH). Interestingly, the population of the evolved RNA replicase displayed a broad distribution of the 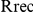 value (Figure 5G; cf. Figure 2), and subsequently diverged into two populations with markedly different distributions of 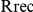 (Figure 5H). Among these two populations, one population had the values of 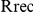 that were significantly higher than the values of 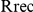 which Rp evolved in the beginning of the simulation (compare Figure 5A and Figure 5H). This population, therefore, resembles the RNA replicase that evolved in the model that included explicitly defined parasites (compare Figure 2D and Figure 5H). By contrast, the other population of the RNA replicase had the values of 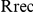 that were lower than the value of 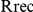 which the Rp evolved in the beginning of the simulation, and it turned out that these values were too small for Rp to survive through self-replication. Thus, this population effectively consisted of parasites. It is most likely that these parasites mediated the coexistence between the RNA replicase and the transcription system.

To summarize, even though this model did not include explicitly predefined parasites as a separate class of molecules and thus did not allow the emergence of parasites with an advantage (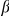) in the recognition by catalysts, the system evolved a DNA replicase and a dual specificity Rp, which then caused the evolution of an effectively parasitic species. Consequently, the system reached an equilibrium state that was essentially identical to the equilibrium state of the model with explicitly predefined parasites. This result demonstrates the robustness of the equilibrium state observed in the original model. Moreover, it elucidates the cause and effect relationship between the evolution of the transcription system and the evolution of parasites. On the one hand, the parasites provide for the evolution of the transcription system by mediating the coexistence with the self-replication system. On the other hand, the transcription system also allows the evolution of the parasites when the model does not include explicitly predefined parasites, by causing the subdivision of the population of the RNA replicase (see the discussion in the next section).

#### Interpretation of the surface model results

The results described above show that the transcription system can evolve in the surface model because the division of labor between the template and the catalyst, which is made possible by the inclusion of DNA molecules in the replication cycle, increases the resistance of the transcription system against parasites compared to the self-replication system.

In addition to this main conclusion, the above results also revealed two general points worthy of note. First, there are two distinct regimes in the stabilization of a certain species by another species, namely, ecological (short-term) stabilization and evolutionary (long-term) stabilization. The results showed that, on the one hand, parasites enabled the transcription system to coexist with the self-replication system. In theoretical ecology, this is known as predator-mediated coexistence [40], [41]. In this regime, the mediation occurs on a short timescale at which each species does not change its character through evolution, hence ecological stabilization. On the other hand, the RNA replicase generated a selection pressure for the transcriptase not to evolve into a dual specificity RNA polymerase. In this regime, a species exerts a selection pressure on the other species so as to maintain its identity on a long timescale at which the other species would have the potential to evolve new features if the stabilizing species was absent, hence evolutionary stabilization.

**Figure 6:**
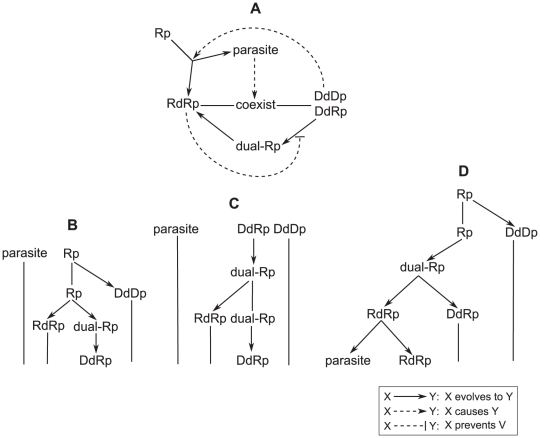
The schematic depiction of the causal (A) and historical (BCD) relationship among the evolution of each species of replicators present at equilibrium in the surface model. Dual-Rp denotes a dual specificity Rp. In B, C and D, the evolutionary dynamics progress from top to bottom. For the explanation, see the main text.

Second, exploration of the model showed that removing any of the three components of the system, namely, the self-replication system, the transcription system and the parasite, at the evolutionary steady state resulted in the restoration of the deleted component through the evolution of the remaining components (Figure 6BCD). Moreover, there was a mutual dependence among the three components with respect to the ecological or evolutionary stability (Figure 6A); e.g., the parasite enabled the evolution of the transcription system through mediating its coexistence with the self-replication system, whereas the transcription system enabled the evolution of the RNA replicase into a parasite-like species. Thus, the causal relationship among the evolutionary fates of the components—i.e. which species causes the evolution of which species—does not form a linear chain, but rather a cycle (Figure 6A). This circularity is in a sharp contrast with the linear structure of the evolutionary history of the species (Figure 6BCD). Therefore, the analysis of an evolutionary trajectory (history) from a single initial condition may not fully elucidate the cause and effect relationships among the evolutionary fates of the species.

#### The flow of information

Given the evolution of the transcription system, it is interesting to consider the separation between template and catalyst in terms of the flows of genetic information. In particular, is the line of descent continued through the replication of DNA in the transcription system? To address this question, we conducted the following experiment. The previous simulation (Table 1, No. 1) was continued after having reached evolutionary equilibrium with the mutation converting Rp into Dp disabled. At the beginning of the simulation, each individual replicator was labeled according to whether it was RNA or DNA. A new individual inherited this label from the template from which it was produced regardless of whether the new individual was RNA or DNA. The simulation was run until the entire populations of the RNA replicase, transcriptase and DNA replicase each descended from either RNA molecules or DNA molecules (not necessarily from one molecule). Then, each molecule was re-labeled, and the simulation was continued: this cycle was repeated 200 times. The result showed that the entire population of the transcriptase was descended from DNA templates of the transcriptase in more than 98% of the simulations. Given that the mean fraction of DNA molecules in the population of the transcriptase was 65%, the origin of this line of descent was significantly biased towards DNA. Likewise, the entire population of the DNA replicase was descended from DNA templates of the DNA replicase in 100% of the simulations whereas the mean fraction of DNA was 78%. These results show that, in the transcription system, the flow of genetic information is unidirectional from DNA to RNA. Therefore, the transcription system established the separation between the template and the catalyst in terms of the flows of genetic information across generations. By contrast, the entire population of the RNA replicases descended from RNA templates in less than 92% of the simulations. Given that the fraction of RNA among the RNA replicases was 95.5% averaged over time, this line of descent was not significantly biased towards RNA templates.

The Central Dogma of molecular biology states that the flow of genetic information is unidirectional from nucleic acids to proteins [42]. In the strict chemical sense, the Dogma is unrelated to the unidirectional flow of information exhibited by the transcription system in the present model. However, the Dogma may be recast in generalized terms, to assert that the flow of genetic information is unidirectional from templates to catalysts. Under this extended interpretation, there seems to be an analogy between the Central Dogma and the unidirectional flow of information from templates (DNA) to catalysts (RNA) exhibited by the transcription system in the present model.

### The compartment model

#### Evolution of a transcription-like system in the compartment model

**Figure 7:**
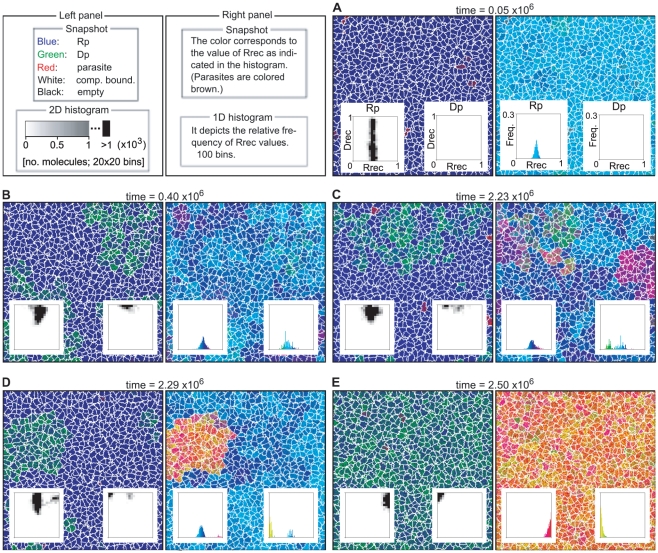
The evolution of the transcription-like system in the compartment model. The compartment model was initialized, and the simulation was run in the same way as in Figure 2. The model was initialized such that the system consisted of a population of Rp enclosed in a compartment. The simulation was first run with the mutation converting Rp into Dp disabled (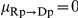). After the system reached evolutionary equilibrium (Figure 7A), the mutation (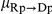) was enabled. The resulting evolutionary dynamics are depicted in panel B to E. The left picture of each panel shows a snapshot of the simulation taken at different times as indicated above panels. The color coding is indicated in the upper left corner of the figure. DNA and RNA are not distinguished. The insets depict two-dimensional histogram of 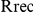 and 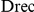. The right picture of each panel shows a snapshot with a different color coding, which indicates the value of 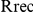. Distinction is not made between Dp and Rp and between DNA and RNA. The insets depict a histogram of 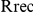 with the same color coding as in the larger pictures that contain them. For the explanation of each panel, see the main text. The parameters were as follows: 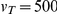 (the volume threshold for division of compartments); 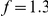 (the target volume is set to the number of internal replicators multiplied by 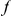); 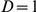; the size of the CA is 512×512 squares; the other parameters were the same as in Figure 2.

As in the surface model, the compartment model was initialized with a homogeneous population of Rp^RNA^ (Table 2, No. 1). The simulation was first run with the mutation converting Rp into Dp disabled. When the system reached equilibrium, Rp displayed a uniform distribution of 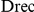
—a trivial consequence of the absence of DNA molecules—and a unimodal distribution of 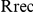 (Figure 7A). The distribution of 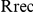 is balanced at some intermediate value by the selection pressure of two opposing directions. The selection pressure at the level of intra-compartment dynamics tends to reduce the value of 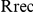 because of the trade-off between templates and catalysts. In contrast, the selection pressure at the level of inter-compartment dynamics tends to increase the value of 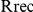 because of the positive coupling between the growth of compartments and the multiplication of internal replicator systems.

**Table 2:** Summary of the results with the compartment models.

No.	Purpose of simulation	Setting of simulation	Ref.	Results
1	Standard simulation. Point of reference	Starting with self-replication system	Fig. 7 & Fig. 8	Transcription-like system evolved. The system was continuously invaded by self-replication system, which always eventually went extinct.
2	To examine if DdDp can maintain DNA templates without reverse transcription	Reverse transcription was suppressed in no. 1 after reaching equilibrium	Text S1, Note 6	DdDp^DNA^ was lost, but DdDp^RNA^ survived via RNA replication by dual-Rp.
3	To examine if the survival of transcription-like system is due to its slower evolutionary deterioration of catalysts	No. 1 was continued with smaller 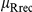 and 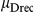 (i.e. slower deterioration)	Text S1, Note 6	Self-replication system out-competed transcription-like system: the advantage of transcription-like system is its slower evolutionary deterioration.
4	The same as in No. 3.	No. 1 is continued with smaller 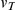		Self-replication system out-competed transcription-like system. See Text S1, Note 6.

**Figure 8:**
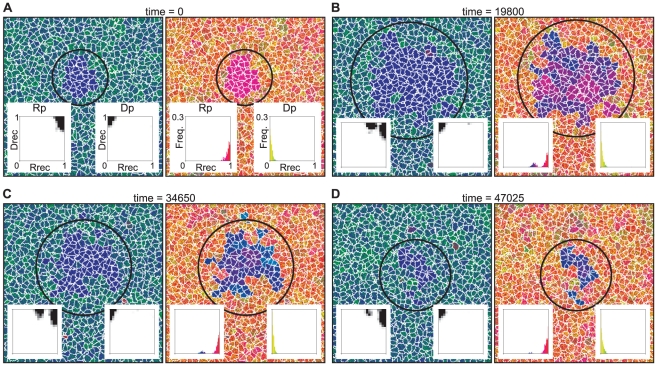
The invasion of compartments containing only Rp^RNA^ and their eventual extinction, which happens repeatedly after the system reached evolutionary equilibrium in the compartment model. The figure depicts the same simulation and in the same format as in Figure 7. The time is reset to zero at an arbitrary moment after the time in Figure 7E. For the explanation of each panel, see the main text.

After the system reached equilibrium, the mutation converting Rp into Dp was enabled. As a result, the Dp with a high activity in DNA replication (a high value of 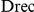) quickly invaded the system (Figure 7B). However, this Dp did not immediately evolve into a DNA replicase, maintaining a moderate reverse transcriptase activity. After the invasion of Dp, the Rp evolved a high transcription activity and a slightly increased RNA replication activity (Figure 7B). The system remained in this state for a long period of time. The existence of Dp at this stage required a continual influx of Dp through mutations because Dp went extinct if the mutation converting Rp into Dp was disabled after the invasion of Dp. Later on, in a subpopulation of compartments, Dp evolved towards decreasing reverse transcription activity (i.e. decreasing the value of 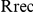) (Figure 7C). Concomitantly, Rp in the same compartments evolved towards increasing RNA replication activity (i.e. increasing the value of 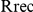) (Figure 7D). As a result, compartments containing a replicator system which consists of a DNA replicase and a dual specificity RNA polymerase appeared in the system (Figure 7D). This replicator system is henceforth referred to as the transcription-like system. The compartments containing the transcription-like system quickly out-competed the other compartments (Figure 7E). After the transcription-like system was established, the model displayed the invasion of compartments that contained only Rp^RNA^, which arose through the chance loss of Dp in compartments containing the transcription-like system (Figure 8A). The compartments containing only Rp^RNA^ quickly increased its population size, locally out-competing the compartments containing the transcription-like system (Figure 8B). However, the compartments containing only Rp^RNA^ displayed the rapid evolution of their internal replicator system, whereby the Rp evolved towards reducing RNA replication activity (Figure 8C). Consequently, the compartments containing only Rp^RNA^ were eventually out-competed by those containing the transcription-like system (Figure 8D). This cycle of invasion and extinction was observed repeatedly. The next section describes why the system displays this complex population dynamics.

#### Compartments containing the transcription-like system experience slower evolutionary deterioration of the internal replicator system than compartments containing only Rp^RNA^

To elucidate the causes of the results described above, the simulation was continued with mutations modifying the values of 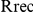 and 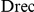 disabled. The result showed that compartments containing only Rp^RNA^ appeared and quickly out-competed compartments containing the transcription-like system. The same result was obtained when the mutation rate was reduced by an order of magnitude (Table 2, No. 3).

The above two results, the repeated cycle of invasion and extinction of compartments containing only Rp^RNA^ and the extinction of compartments containing the transcription-like system under sufficiently reduced mutation pressure, have two implications. Firstly, compartments increase their fitness by losing the DNA replicase (i.e., when Rp^RNA^ remains the only catalyst in a compartment). Secondly, however, compartments containing only Rp^RNA^ experience faster evolutionary deterioration of the internal replicator system than compartments containing the transcription-like system, an effect that confers a selective advantage onto the compartments containing the transcription-like system under sufficiently strong mutation pressure.

To investigate the first effect, we directly measured the fitness of compartments as follows. The model was modified to make the boundaries of the compartments completely unchangeable so the model contained compartments but no compartment dynamics. The model was initialized in a configuration where the size of the compartments was equal to the threshold above which compartments divide in the original model (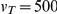). Two simulations were conducted. In the first simulation, compartments were filled with a population of Rp^RNA^, Rp^DNA^, Dp^RNA^ and Dp^DNA^ in equal proportion. The values of 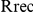 and 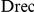 were set such that the transcription-like system was established (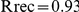 and 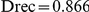 for Rp; 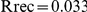 and 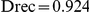 for Dp—these values were obtained from the system depicted in Figure 7E). In the second simulation, compartments were filled with a population of Rp^RNA^ alone (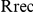 and 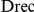 were the same as before). The simulations were run with all mutations disabled (all the other parameters were the same as in Figure 7). We then measured the rate at which the compartments lost all internal replicators (i.e. the death rate of compartments) and the density of the replicators in the surviving compartments (directly related to the growth rate of compartments). The result was that the compartments indeed increased fitness by losing the DNA replicase (for the compartments containing the transcription-like system, the death rate was 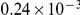, and the average density of internal replicators was 0.81; for the compartments containing only Rp^RNA^, the death rate was 0 within the timescale of measurement, and the average density of internal replicators was 0.94). This result is understandable because, as discussed above, the inclusion of DNA in a replication cycle leads to a fourfold increase in the number of the types of molecules required for multiplication and so reduces the efficiency of multiplication and increases the chance of stochastic loss of essential molecules. Therefore, compartments increase their fitness by losing the DNA replicase.

To assess the second effect, we compared the evolutionary deterioration rates between the self-replication system and the transcription-like system (Table 4, No. 1 and 2). To simplify the comparison, we considered a large, well-mixed replicator system. The greater size of the replicator system reduced the effect of random drift in the population dynamics of replicators and so allowed us to focus on the deterministic aspect of the evolutionary deterioration process over a sufficiently long time (note that selection pressure is a deterministic factor). To this end, we modified the model such that interactions between molecules occur globally (i.e. interactions can occur independent of the locations of molecules) so that the system was effectively well-mixed. Again, two simulations were conducted. In one simulation, the system was initialized with a population of Rp^RNA^, Rp^DNA^, Dp^RNA^ and Dp^DNA^ in equal proportion. The value of 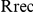 and 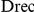 were set such that the replicator system was the idealized, pure transcription-like system (to be precise, 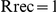 and 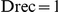 for Rp; 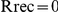 and 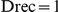 for Dp). In the other simulation, the system was initialized with a population of Rp^RNA^ alone (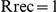 and 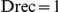).

**Figure 9:**
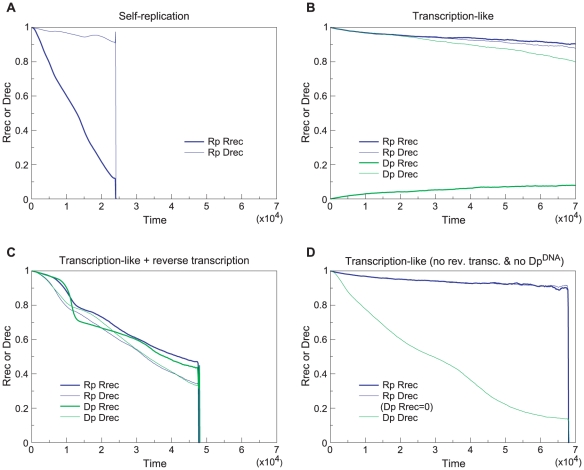
The time course of evolutionary deterioration of catalysts under well-mixed condition with a large population size for various replication systems. The model was modified such that interactions between molecules happen globally regardless of the location of molecules (the system is effectively well-mixed). The model was initialized with a population of Rp^RNA^ in panel A, with a population of Rp^RNA^, Rp^DNA^, Dp^RNA^ and Dp^DNA^ in equal proportion in panel B and C, and with a population of Rp^RNA^, Rp^DNA^ and Dp^RNA^ in equal proportion in panel D. The initial value of Rrec and Drec were set as indicated in the figure (at time = 0). The parameters were as follows: 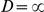
(effectively); the size of the CA is 512×512 squares; the other parameters were the same as in Figure 2.

The result was that the system consisting only of Rp^RNA^ rapidly deteriorated, with the value of 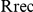 quickly decreasing, and eventually went extinct (Figure 9A). The 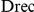 of Rp^RNA^ underwent neutral evolution because of the absence of DNA molecules (Figure 9A). This rapid deterioration of the replication activity is expected because it is selectively disadvantageous to be a catalyst in a well-mixed self-replication system with complex formation [22]. In contrast, the transcription-like system displayed evolutionary deterioration that was qualitatively indistinguishable from neutral evolution (compare Figure 9B and Figure 9A). Although the transcription-like system also went extinct eventually, this took much longer time than for the self-replication system (time≈10^5^). This qualitative difference in the rates of evolutionary deterioration of catalysts supports the argument that compartments containing the transcription-like system experience slower evolutionary deterioration of the internal replicator system (the origin of this difference will be elucidated in the next section).

If the advantage of compartments containing the transcription-like system lies in the slower evolutionary deterioration of internal replicator systems, it is expected that altering the severity of evolutionary deterioration would modulate the evolvability of the transcription-like system. Decreasing the mutation rate of replicators, obviously, delays evolutionary deterioration. Thus, if the mutation rate is sufficiently reduced, the advantage of the transcription-like system must be so insignificant that compartments containing the transcription-like system are unable to out-compete those containing only Rp^RNA^. In fact, this has already been seen in one of the simulations described above (Table 2, No. 3). Moreover, the severity of evolutionary deterioration also depends on the population size of the internal replicator system (i.e. the size of compartments, 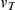) because the population size determines the level of random drift in the evolutionary dynamics, which disturbs the deterministic force of selection and, consequently, generates greater variation among the compartments, on which the compartment-level selection operates. Thus, if the size of compartments is smaller, the evolutionary deterioration of the internal replicator system should be slower [35]. Therefore, it is expected that, if the size of compartments is sufficiently small, compartments containing the transcription-like system are unable to out-compete those containing only Rp^RNA^. These expectations were indeed confirmed by additional simulations (see Text S1, Note 6, for details).

To summarize, the transcription-like system can confer both advantage and disadvantage to a compartmentalized replicator system compared to the self-replication system: it impedes the evolutionary deterioration of the internal replicator system but hampers the efficiency of multiplication of that system. The disadvantage due to the reduced efficiency of multiplication is significant regardless of the parameter values because it is a necessary consequence of an increase in the complexity of replication cycle brought about by the inclusion of DNA. In contrast, the advantage due to the slower evolutionary deterioration depends on how fast the evolutionary deterioration proceeds if compartments contain the self-replication system, which, in turn, depends on the mutation rate of individual replicators and the size of compartments (or more precisely, the population size of the internal replicator system). If the rate of the evolutionary deterioration of compartments containing the self-replication system is sufficiently high, the advantage of the transcription-like system more than compensates for the disadvantage, and the evolution of the transcription-like system becomes possible.

#### The presence of transcription and the absence of reverse transcription prevents the evolutionary deterioration of catalysts in the internal replicator system

We next consider the reason why, in the compartment model, the transcription-like system displays slower evolutionary deterioration than the self-replication system. As already mentioned, in the self-replication system, catalysts gain a selective advantage by decreasing the time they spend replicating templates (i.e. by decreasing the value of 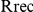 and 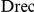) because of the trade-off between templates and catalysts. By contrast, in the transcription system, the catalysts do not function as templates, so there is no selective advantage for catalysts to reduce the time spent on replicating templates. However, in the transcription-like system, Rp maintains a high RNA replication activity, which raises the question whether this impairs the release from the trade-off.

To address this question, we consider an ordinary differential equation (ODE) which simulates the population dynamics of the internal replicator system of compartments in the compartment model (Table 3). Although the ODE model does not fully reflect the evolutionary dynamics of the internal replicator system, it captures the deterministic aspect of the dynamics under idealized conditions where random drift and mutations play no role. This simplification makes it easier to investigate the deterministic stability of replicator systems. The first ODE model we constructed described the population dynamics of one species of RNA polymerase and one species of DNA polymerase (see the equation in Text S1). The strategy of the analysis was to use the idealized transcription system (i.e. 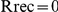 and 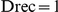 for both Rp and Dp) as a reference point and then consider the effect of adding RNA replication (i.e. setting 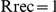 for Rp) and, for the purpose of comparison, reverse transcription (i.e. setting 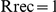 for Dp) to the transcription system.

**Table 3:** Summary of the results with the ODE models.

No.	Purpose of simulation	Setting of simulation	Results
1	Point of reference	Transcription system (i.e. 1 species of DdRp and 1 species of DdDp).	The system survived. (The ratio of Rp^RNA^ to Dp^RNA^ and that of Rp^DNA^ to Dp^DNA^ was determined by the initial ratio of Rp^DNA^ to Dp^DNA^.)
2	To examine the effect of adding RNA replication activity on the stability of transcription system (No. 1)	Transcription-like system (i.e. 1 species of dual-Rp and 1 species of DdDp)	The system survived: the addition of RNA replication activity to transcription system did not impair the survival of the system.
3	To examine the effect of adding reverse transcription activity on the stability of transcription system (No. 1)	Transcription system+reverse transcription activity (i.e. 1 species of DdRp and 1 species of dual-Dp)	The system went extinct: the addition of reverse transcription activity to transcription system impaired the survival of the system.
4	To examine if it is selectively advantageous for catalysts to lose catalytic activity in transcription-like system (No. 2)	Transcription-like system (as in No. 2) + mutant Rp	The system survived even if 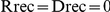 for mutant Rp: losing catalytic activity is not selectively advantageous for catalysts in transcription-like system.

The ODE model was numerically solved for various initial conditions. The results showed that the transcription system could survive under well-mixed conditions if the initial ratio of Rp^DNA^ to Dp^DNA^ was neither too large nor too small (Table 3, No. 1). In the transcription system, there is symmetry between Rp and Dp because of the assumption that catalysts do not discriminate between different templates of the same molecular type. Because of this symmetry, the steady state ratio of Rp^DNA^ to Dp^DNA^ and that of Rp^RNA^ to Dp^RNA^ are determined by the initial ratio of Rp^DNA^ to Dp^DNA^ (i.e., there is structural instability in the system).

We next considered the effect of adding either RNA replication or reverse transcription to the transcription system. Before describing the results, it is worth noting that adding either of these processes introduces asymmetry into the system. For instance, adding RNA replication makes Rp^RNA^ play both the role of catalysts and templates *for RNA replication*, whereas Dp^RNA^ plays only the role of templates. Of course, Dp^RNA^ also plays the role of catalysts for DNA replication, but so does Rp^RNA^ for transcription—there is no asymmetry between Dp and Rp with respect to reactions involving DNA molecules. Due to the above asymmetry, Dp^RNA^ will be replicated by Rp^RNA^ more often (per molecule) than Rp^RNA^ is replicated by Rp^RNA^ assuming that the initial condition is symmetric with respect to Dp and Rp. This can be seen from the fact that three times more Rp^RNA^ is required than Dp^RNA^ in order to produce an equal amount of the two complex molecules (i.e. that between Rp^RNA^ and Rp^RNA^ and that between Rp^RNA^ and Dp^RNA^) [22]. It is easily seen that adding reverse transcription likewise introduces asymmetry into the transcription system.

The results of the numerical calculation indicated that adding RNA replication did not adversely affect the survival of the system (Table 3, No. 2). In contrast, adding reverse transcription led to the extinction of the system (this was the case even when the value of 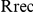 was very small, e.g. 0.01; Table 3, No. 3). To determine the origin of this difference, we searched for a factor(s) that dampened the asymmetry generated by RNA replication and a factor(s) that amplified the asymmetry generated by reverse transcription. Let us first consider the case of reverse transcription. If there is no reverse transcription, the initial condition determines the ratio of Rp^DNA^ to Dp^DNA^ which itself determines the ratio of Rp^RNA^ to Dp^RNA^. Let us suppose that the system is initially symmetrical between Rp and Dp. Now, by adding reverse transcription, Rp^DNA^ is produced at a slightly higher rate than Dp^DNA^ due to the aforementioned asymmetry in reverse transcription, so that the ratio of Rp^RNA^ to Dp^RNA^ increases through transcription, which, in turn, leads to the further increase of the Rp^DNA^ to Dp^DNA^ ratio, hence an amplifying factor. Next, for the case of RNA replication, assuming a symmetric initial condition again, adding RNA replication slightly increases the ratio of Dp^RNA^ to Rp^RNA^. However, the change of the Dp^RNA^ to Rp^RNA^ ratio has no effect on the Dp^DNA^ to Rp^DNA^ ratio because there is no reverse transcription. Thus, transcription tends to bring the Dp^RNA^ to Rp^RNA^ ratio back to the Dp^DNA^ to Rp^DNA^ ratio, hence a dampening factor. To summarize, provided the absence of reverse transcription, the transcription of DNA molecules tends to bring the population composition of RNA molecules towards that of DNA molecules and so dampens the bias in the population composition of RNA molecules generated by the asymmetry in RNA replication. In other words, the population of DNA molecules serves as a buffer to the population of RNA molecules.

The above argument implies that in the absence of reverse transcription, transcription prevents the evolutionary deterioration of catalysts because the selective advantage a catalyst would obtain by increasing the time it spends being a template is effective only in the RNA population, and such a selective advantage would be counteracted by the transcription of DNA molecules. To examine whether this is indeed the case, we extended the ODE model to include the population of an additional species of Rp, which we refer to as Rp′. The strategy of the analysis was to treat Rp′ as a mutant of Rp and examine whether Rp′ could out-compete the original Rp when the small amount of Rp′ was introduced into the system, which mimicked the mutation of Rp into Rp′. The value of 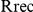 and 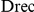 were set to yield the idealized transcription-like system (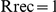 and 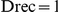 for Rp, and 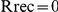 and 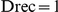 for Dp). The system was initialized such that it was symmetrical with respect to Rp and Dp, and the population size of Rp′ (both RNA and DNA) was set to 0. After the system reached equilibrium, the population size of Rp′ (both RNA and DNA) was increased by a small amount (0.001), and the system was allowed to reach a new equilibrium. The result showed that even if Rp′ completely lost its catalytic activity (i.e. 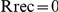 and 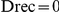), Rp′ was unable to out-compete Rp (the population size of Rp′ remained small; Table 3, No. 4). Thus, in the absence of reverse transcription, transcription impedes the evolutionary deterioration of catalysts.

The above argument shows that the release of catalysts from the template-catalyst trade-off depends not only on the presence of transcription but also on the absence of reverse transcription, i.e. blockage in the flow of information from catalysts (RNA) to templates (DNA). To investigate this issue, we conducted a simulation of the type shown in Figure 9, i.e. the examination of evolutionary deterioration process in a large, well-mixed replicator system (Table 4, No. 3). The system was again initialized with the transcription-like system; however, this time, reverse transcription activity was added to the system (i.e. the 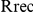 of Dp was set to 1). The result of this simulation showed that addition of reverse transcriptase greatly accelerates the evolutionary deterioration of catalysts (Figure 9C).

**Table 4:** Summary of the results with the well-mixed CA model.

No.	Purpose of simulation	Setting of simulation	Fig.	Results
1	To measure the speed of evolutionary deterioration of catalysts in self-replication system	Self-replication system (only RdRp^RNA^) with 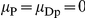	9A	RdRp quickly evolved towards catalytic deterioration.
2	To measure the speed of evolutionary deterioration of catalysts in transcription-like system	Transcription-like system (dual-Rp + DdDp) with 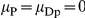	9B	The deterioration of catalysts was as slow as neutral evolution.
3	To examine the effect of adding reverse transcription activity on the speed of the evolutionary deterioration of catalysts in transcription-like system (No. 2)	Transcription-like system+reverse transcription (dual-Rp+dual-Dp) with 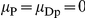	9C	Reverse transcription speeded up the evolutionary deterioration of catalysts.
4	The effect of the absence of Dp^DNA^ on the speed of the evolutionary deterioration of Dp in transcription-like system (No. 2)	Transcription-like system without Dp^DNA^ with reverse transcription suppressed and with 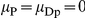	9D	The absence of Dp^DNA^ speeded up the evolutionary deterioration of Dp.

#### Information flow

As shown above, breakage in the flow of information from RNA to DNA (elimination of reverse transcription) is an important factor underlying the advantage of the transcription-like system in a compartmentalized replicator system. However, although reverse transcription activity was much reduced in the transcription-like system, it was not completely absent due to the mutation pressure. Moreover, the transcription-like system maintained a high rate of RNA replication (Rp was dual specific). Therefore we were interested to find out in which direction the information was transmitted among replicators in the long run: from RNA to DNA, or from DNA to RNA, or both? To address this question, we conducted the same simulation as for the surface model in order to trace the line of descent over generations. This simulation showed that the population of the dual specificity Rp was always descended from its DNA templates (i.e. Rp^DNA^). Therefore, from the actual flows of genetic information, the division of labor between the template and the catalyst was established for the dual specificity Rp despite the fact that it maintained a high RNA replicase activity. However, the situation differed for the DNA replicase. The simulation showed that two populations of compartments quickly arose in the system: in one of these populations, the Dp was descended from Dp^DNA^, whereas in the other population the Dp was descended from Dp^RNA^. Because of the finiteness of the system, the entire population of Dp was eventually descended either from Dp^RNA^ or from Dp^DNA^, with the choice determined by chance.

To further examine the case of Dp, we completely removed reverse transcription activity from the model (i.e. the value of 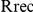 for Dp and its mutation rate were set to zero). The simulation showed that Dp^DNA^ was eventually lost from the system whereas Dp^RNA^ remained (Table 2, No. 2). This is possible because Dp^RNA^ can be amplified through RNA replication by the dual specificity Rp. In the absence of Dp^DNA^, the equilibrium value of 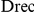 of Dp was slightly decreased (data not shown). This seems to occur because Dp^RNA^ was maintained through RNA replication, so the trade-off between template and catalyst set in and caused selection pressure on Dp^RNA^ towards decreasing 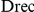. This interpretation was supported by a simulation of the type shown in Figure 9, i.e. examination of evolutionary deterioration in a large, well-mixed replicator system (Table 4, No. 4). The system was initialized with the transcription-like system without Dp^DNA^, and reverse transcription activity was completely removed in the same way as above. This simulation showed that Dp evolved towards decreasing DNA replication activity much faster than did the transcription-like system (Figure 9D; compare with Figure 9B). This result gives further support to the conclusion that DNA molecules can prevent or at least slow down the evolutionary deterioration of catalysts. Moreover, this simulation shows that the survival of Dp^RNA^ does not require the existence of Dp^DNA^ (because of dual-specificity Rp), which can explain why the line of descent for Dp was not always continued through DNA replication in the original compartment model (Table 2, No. 2). (See Text S1, Note 7, for additional discussion.)

### Models without complex formation

The preceding sections argued that the evolution of DNA-like molecules is driven by the ability of the division of labor between the template and the catalyst to eliminate the advantage of parasites originating from the trade-off between template and catalyst. To further test this argument, we removed the complex formation from the models and instead assumed that the replication reaction is an instantaneous process: 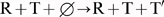. This is expected to significantly reduce the effect of the trade-off (but see below). Examination of the models without complex formation showed that Dp did not evolve under various parameter combinations (Table 1, No. 7; Table 2, No. 5). This result is in accord with the above argument. However, deviation from this outcome was observed under conditions that were not considered in the original models. Specifically, in the surface model without complex formation, if the decay rate of DNA was substantially lower than that of RNA and if the diffusion rate 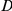 was sufficiently low, Dp could be evolutionarily maintained (i.e. Dp survived if the system initially consisted of the transcription system and the self-replication system, but it could not evolve if the system initially consisted of the self-replication system only). In the compartment model without complex formation, Dp evolved when the diffusion of molecules across compartment boundaries was enabled and the system included an explicitly predefined parasite. However, it has to be kept in mind that assuming instantaneous replication does not completely remove the trade-off between template and catalyst in RNA-like replicators because, if a catalyst replicates other templates, this leads to local depletion of the resource under finite diffusion, decreasing the chance of the catalyst itself being replicated. This interpretation was supported by analysis of the compartment model in which the complex formation was removed and compartment boundaries were immobilized. In this model, when 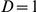 (diffusion), which was the value used in the compartment models to achieve a relatively well-mixed condition in the internal replicator system, the self-replication system evolved towards decreasing RNA replication activity. Thus, assuming instantaneous replication is not a perfect control experiment with respect to the trade-off between template and catalyst. Nevertheless, the finding that Dp did not evolve in the model without complex formation under the conditions considered in the original models (i.e. no difference between DNA and RNA other than the presence-absence of catalytic ability and no diffusion across compartment boundaries) implies the importance of the trade-off enhancement by complex formation for the evolution of DNA.

## Discussion

It has been customarily assumed that the evolution of DNA should be explained by some advantageous properties of DNA as template, e.g., the higher stability of DNA compared to RNA. However, the current study shows that, in RNA-like replicator systems, the lack of catalytic activity in DNA-like molecules in itself can give rise to a selection for the emergence and fixation of DNA molecules. In the surface model, DNA allowed the evolution of the division of labor between the template (DNA) and the catalyst (RNA), which mitigated the adverse effect of parasites arising from the trade-off between templates and catalysts. In the compartment model, DNA could cause the retardation of the evolutionary deterioration of the internal replicator system of compartments by eliminating the advantage of RNA molecules being non-catalytic, i.e. evolving into parasites, which originated from the aforementioned trade-off. This retardation required the presence of transcription and the absence of reverse transcription. In other words, the information must flow from DNA-like molecules (template) to RNA-like molecules (catalyst) but not vice versa. This unidirectionality of the information flow is also a form of division of labor between the template and the catalyst. Therefore, both models effectively yield the same conclusion: DNA can nullify the disadvantage of RNA functioning as a catalyst—and hence the advantage of parasites—through establishing the division of labor between the template (DNA) and the catalyst (RNA). This advantage can more than compensate for the disadvantage due to the reduced efficiency of multiplication caused by the increased complexity of the replication cycle.

Although the transcription system avoids the trade-off between template and catalyst through establishing the division of labor between the template and the catalyst, it generates the trade-off between replication and transcription whereby a template (DNA) must spend a part of its lifetime being transcribed in order to produce catalysts (RNA), and during these times, the template cannot be replicated. The latter trade-off causes a selection pressure for templates to evolve towards decreasing the rate of transcription in exchange for increasing the rate of replication. In the present models, however, this selection pressure does not affect the evolution because the models do not allow templates to evolve their affinities towards Rp and Dp so as to differentiate between replication and transcription. To examine the effect of the replication-transcription trade-off on the evolution of DNA, we slightly modified the models to allow templates to distinguish between Rp and Dp (see Text S1 for details). The results showed that, although the effect of the trade-off between replication and transcription was non-negligible as the models exhibited the evolution of templates to reduce transcription, it was not large enough to qualitatively change the main results obtained with the original models. This result corresponds to a well-known fact from the group selection theory [43] that the condition required for the evolution of “weak altruism” (the action that is beneficial to the individual that performs it but gives greater benefits to the other individuals of the same “group”) is much less strict than the condition required for the evolution of “strong altruism” (the action that gives no benefit but a cost to the performer of the action). Thus, everything else being equal, the selection against strong altruism is stronger than the selection against weak altruism. Indeed, in the trade-off between template and catalyst, when a catalyst replicates templates, this gives no benefit to the catalyst itself and so corresponds to strong altruism. By contrast, in the trade-off between replication and transcription, when a template (DNA) is transcribed, this gives a benefit not only to the other templates but also to the template that is transcribed through the production of catalysts, hence weak altruism. Therefore, the suppression of the template-catalyst trade-off should more than compensate for the generation of the transcription-replication trade-off.

Although the order of appearance of different types of biopolymers during primordial evolution is still debated [44], [45], the universality of the translation machinery in all domains of life suggests that proteins most likely evolved in the RNA world before DNA (e.g., [46]). If RNA molecules functioned predominantly as templates in the RNA-protein world, the division of labor between templates and catalysts was established before the emergence of DNA. The basic tenet of the present study, namely, that dedicated templates (DNA) can release catalysts (RNA) from the trade-off between template and catalyst through establishing the division of labor between templates and catalysts, seems to be also applicable to the evolution of proteins in the RNA world. Indeed, the relegation of the catalytic functions to proteins so that RNA molecules turn into dedicated templates might achieve an effect similar to the effect of the separation of functions between DNA (template) and RNA (catalyst) in the present models. In the RNA-protein world, the trade-off between RNA replication and RNA translation becomes relevant as the same RNA molecule is used both for replication and for translation. However, this trade-off implies weak altruism as opposed to the strong altruism implicit in the template-catalyst trade-off, so the separation of functions is likely to be beneficial for the replicator system (see above).

The question arises whether there could be advantages associated with the emergence of DNA (irrespective of its chemical properties) in the RNA-protein world. In this case, DNA can release RNA from the trade-off between replication and translation so that RNA can be dedicated to translation. This effect might cause a substantial reduction in the selective advantage of parasitic templates because the suppression of RNA replication due to translation would be more severe than the suppression of DNA replication due to transcription assuming equal rates of protein production (the rate of DNA transcription can be smaller than that of RNA translation, so DNA transcription would impede DNA replication less than RNA translation impedes RNA replication). Moreover, if a high rate of protein production is selectively advantageous to the system, releasing RNA from the replication function and so allowing it to be dedicated to translation might be a substantial advantage to the system, causing strong selection pressure for the evolution of DNA.

The present models assume that Dp can emerge from Rp through a one-step mutation. This simplification was made because the central question of the current study was whether there could be any selective advantage for an RNA-based evolving system to produce DNA-like molecules independent of specific nucleic acid chemistry. It appears that our main conclusion on the existence of such a selective advantage should be valid independent of specific assumptions on the mutation. To further assess the validity of this conclusion, we also investigated the models under two different assumptions on the effects of mutations. Under the first assumption, the distinction between Rp and Dp was continuous. Each replicase is assigned two parameters that determine the product specificity: 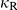 and 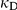, the rate constants of RNA and DNA production, respectively. The ratio 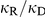 assumed non-negative values with the constraint that 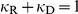 and could be modified by mutations (in the original model, this ratio was either 0 or infinite). Under this assumption, DNA evolved in both the surface model and the compartment model. The population of catalysts consisted of only one species that catalyzed both RNA and DNA production. Moreover, the surface model displayed the evolution of both product and template specificity toward DNA: 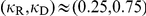 and 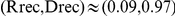. In contrast, the compartment model displayed only the evolution of template specificity toward DNA—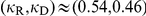 and 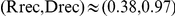, probably because of the between-compartment selection that tends to increase the number of catalysts (RNA) within compartments. Under the second assumption on the mutation, a replicase was either Rp or Dp as in the original model, but there was a continuous range of catalytic capacity associated with each polymerase: 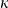 assumed a value between 0 and 1 and could be modified by mutations. When Rp mutated into Dp (through a one-step mutation), the value of 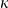 was set to 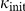. When 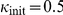, the surface model showed qualitatively the same result as the original model, whereas the compartment model did not display the evolution of DNA—a result indicating the greater robustness of the results with the surface model. To summarize, these experiments with (partially) continuous mutation effect models revealed the evolution of DNA and so appear to validate our main conclusion on the intrinsic advantage of the template-catalyst separation.

Kaneko and Yomo [47] proposed that molecules must be a minority to display hereditary properties in a protocell (see [47], for the exact meaning of “hereditary properties”). In their study, it is conceived that the hereditary molecule emerges not as a result of Darwinian evolution due to the selective advantage it confers to a protocell but as a physical consequence of factors that are not necessarily related to the hereditary properties (such as higher-order catalysis and kinetic asymmetry). By contrast, in the present compartment model, DNA evolved due to a selective advantage conferred to the respective protocells (compartments). Moreover, DNA molecules constituted 30–40% of the total population of internal replicators and so were not a small minority.

Zintzaras et al [48] investigated the consequence of the trade-off between catalytic activity and template affinity to RNA polymerase in ribozymes and proposed that the complete absence of competition between two species of ribozymes could lead to the evolutionary divergence where one species functions slightly more efficiently as a catalyst and the other functions more efficiently as a template. Despite the superficial similarity, this form of divergence crucially differs from the division of labor between template and catalyst discussed here. In the model of Zintzaras et al., the two species were *not* templates for each other, so both ribozymes must function as templates to transmit information to subsequent generations. In the present models, DNA allows RNA (ribozyme) to not function as template at all and releases it from the template-catalyst trade-off. In addition, the division of labor between template and catalyst evolved without assuming reduced competition between replicators.

We previously noted an interesting difference between the concepts of genotype as applied to modern cells and protocells conceived as vesicle-like compartments containing replicators [35]. A common assumption is that the genotype of an individual is static on the timescale of an individual's lifetime. Although valid for modern cells, this assumption might be invalid for the protocell because the internal replicator system of a protocell—the population of which can be viewed as the genome—undergoes evolutionary deterioration over time comparable to the lifetime of a compartment due to the within-compartment selection [35]. The current study has shown that the division of labor between template and catalyst can prevent such rapid evolutionary deterioration. The evolutionary stabilization of the internal replicator system caused by DNA can be considered a step toward the evolution of the modern-type, relatively stable genotype in protocells.

## Supporting Information

Text S1 The text contains notes to the main text, the implementation details of the surface model and the compartment model, the ODE models and the description of the models that take account of the trade-off between replication and transcription.
(PDF)
