# Supplementary material for: On the Origin of DNA Genomes: Evolution of the Division of Labor between Template and Catalyst in Model Replicator Systems
Source: PLoS Comput Biol. 2011 Mar 24;7(3):e1002024. doi: 10.1371/journal.pcbi.1002024 (PMC3063752; doi:10.1371/journal.pcbi.1002024)
Supplement: Text S1 — The text contains notes to the main text, the implementation details of the surface model and the compartment model, the ODE models and the description of the models that take account of the trade-off between replication and transcription. (PDF) [file pcbi.1002024.s001.pdf]

Supporting Information: Text S1

Notes to the Main Text

1. The population mean of the Rrec values at evolutionary equilibrium was an increasing function of β; i.e., if the parasite is more competitive as a template and therefore can exploit the catalyst more severely, the catalyst evolves towards recognizing templates better. This dependence probably emerges from the fact that, when the parasite is highly competitive, the wave front must progress faster in order to stabilize traveling waves, and more stable waves will be selected (for more on the selection on the level of traveling waves, see [1]).

2. As mentioned under the "Results" section, the requirement of the joint action of the four types of molecules reduces the multiplication rate of the transcription system. This effect is especially enhanced when the diffusion of molecules is limited as is the case in the surface model. Finite diffusion and the locality of replication yielded a correlation (co-localization) between the spatial distributions of templates and their products. Specifically, $\mathrm{Rp}^{\mathrm{DNA}}$ (template) and $\mathrm{Rp}^{\mathrm{RNA}}$ (product), and $\mathrm{Dp}^{\mathrm{DNA}}$ (template) and $\mathrm{Dp}^{\mathrm{RNA}}$ (product) are co-localized. This effect causes strong positive feedback in the production of $\mathrm{Dp}^{\mathrm{DNA}}$, resulting in its overabundance, apparently, because the product ($\mathrm{Dp}^{\mathrm{DNA}}$) of the reaction between $\mathrm{Dp}^{\mathrm{DNA}}$ and $\mathrm{Dp}^{\mathrm{RNA}}$ (DNA replication) gives feedback only to this reaction, whereas the product ($\mathrm{Rp}^{\mathrm{RNA}}$) of the reaction between $\mathrm{Rp}^{\mathrm{RNA}}$ and $\mathrm{Rp}^{\mathrm{DNA}}$ (transcription) gives feedback not only to this reaction but also to the production of $\mathrm{Dp}^{\mathrm{RNA}}$ through transcription, which, in turn, promotes the production of $\mathrm{Dp}^{\mathrm{DNA}}$ preferentially over $\mathrm{Rp}^{\mathrm{DNA}}$ due to the spatial co-localization between $\mathrm{Dp}^{\mathrm{RNA}}$ and $\mathrm{Dp}^{\mathrm{DNA}}$. The overabundance of $\mathrm{Dp}^{\mathrm{DNA}}$ results in the occasional loss of $\mathrm{Rp}^{\mathrm{RNA}}$ from local areas, which causes local extinction (Figure 3, encircled regions). This inefficiency of multiplication results in the patchiness of the spatial distribution of replicators.

3. A previous study on a self-replication system has shown that the scarcity of catalysts, which reduces the chance of a template encountering catalysts, affects the survival of the parasites more severely than the survival of the catalyst. This is because the co-localization among replicators of the same line of descent decreases the chance of a parasite to encounter a catalyst relative to the chance of a catalyst to encounter another catalyst [2]. The same argument should, in principle, be applicable to the current model, where the overproduction of $\mathrm{Dp}^{\mathrm{DNA}}$ results in the scarcity of catalysts (see Text S1, Note 2).

4. We conducted an additional simulation in which reverse transcription was suppressed after the system reached evolutionary equilibrium by setting the Rrec of the DNA replicase to zero and disabling the mutation on this parameter (note that reverse transcription activity was not completely absent in the original simulation because the mutation constantly modifies the value of Rrec). In this simulation, the absence of the self-replication system did not lead to the evolution of the transcriptase into a dual specificity Rp (Table 1, No. 5). Thus, the self-replication system is not required for the evolutionary stabilization of the transcription system when reverse transcription activity is completely suppressed. This result implies a causal link between the evolutionary destabilization of the transcription system and the presence of weak reverse transcription activity in the system.

5. This was probably due to the selective disadvantage of catalysts arising from the trade-off between template and catalyst as explained above and due to the lack of selection at the level of traveling waves (see also Text S1, Note 1).

6. Decreasing the size of compartments slows down the evolutionary deterioration of the internal replicator system of compartments. This is supported by the result that, in the system with

compartments containing only Rp, Rrec at evolutionary equilibrium was a decreasing function of the size of compartments.

If the size of compartments is sufficiently small, compartments containing the transcription-like system are unable to out-compete those containing only $\mathrm{Rp}^{\mathrm{RNA}}$. This was confirmed by the competition experiments with smaller values of v_T (threshold for division) for the same mutation rate, which showed that compartments containing the transcription-like system were out-competed by those containing only $\mathrm{Rp}^{\mathrm{RNA}}$ when $v_T \leq 300$ (Table 2, No. 4).

7. As can be seen from the comparison between Figure 9A and Figure 9D, the evolutionary deterioration of Dp in compartments containing the transcription-like system without $\mathrm{Dp}^{\mathrm{DNA}}$ (Table 4, No. 4) was significantly slower than the deterioration of Rp in compartments containing the self-replication system (Table 4, No. 1). This difference can be explained in terms of the difference in the "directness" of the trade-off between template and catalyst. In the case of the self-replicating $\mathrm{Rp}^{\mathrm{RNA}}$, the trade-off is direct, in that the interactions in which $\mathrm{Rp}^{\mathrm{RNA}}$ is potentially a catalyst are the same as those in which $\mathrm{Rp}^{\mathrm{RNA}}$ is potentially a template (i.e. the interactions between two molecules of RpRNA). In the case of $\mathrm{Dp}^{\mathrm{RNA}}$, the trade-off is indirect, in that the interactions in which $\mathrm{Dp}^{\mathrm{RNA}}$ is potentially a catalyst (i.e. the interactions between $\mathrm{Dp}^{\mathrm{RNA}}$ and DNA) are distinct from the interactions in which $\mathrm{Dp}^{\mathrm{RNA}}$ is potentially a template (i.e. the interactions between $\mathrm{Dp}^{\mathrm{RNA}}$ and $\mathrm{Rp}^{\mathrm{RNA}}$). Because of this difference, $\mathrm{Dp}^{\mathrm{RNA}}$ obtains a smaller advantage from reducing its catalytic activity than $\mathrm{Rp}^{\mathrm{RNA}}$.

The Details of the CA Models

The Surface Model

The replicator dynamics specified in the main text was modeled in stochastic cellular automaton (CA) framework. The model is a spatially extended, individual-based, Monte Carlo simulation model. It consists of a two-dimensional square grid and molecules located on the grid. One square in the grid can contain at most one molecule, which is either $\mathrm{Rp}^{\mathrm{RNA}}$ or $\mathrm{Rp}^{\mathrm{DNA}}$ or $\mathrm{Dp}^{\mathrm{RNA}}$ or $\mathrm{Dp}^{\mathrm{DNA}}$ or $\mathrm{parasite}^{\mathrm{RNA}}$ or $\mathrm{parasite}^{\mathrm{DNA}}$; empty square is considered as the generalized resource for replication (i.e. $\emptyset$ in $\mathrm{C} + \emptyset \rightarrow \mathrm{R} + \mathrm{T} + \mathrm{T}'$). A complex molecule ($C$) consists of two molecules, which must always be located in contiguous squares. Contiguity is defined as the eight nearest squares (Moore neighbors). The state of the CA is fully specified by the spatial distribution of molecules with "flags" specifying which molecules form complexes. The temporal dynamics of the model was run by consecutively applying the stochastic algorithm that simulated reactions—namely complex association, complex dissociation, replication and decay—and diffusion.

The reaction-diffusion algorithm effectively runs as follows:

1. Randomly choose one square from the CA (every square with an equal probability).

2. Determine the possbile reactions and their rate constants as follows. Depending on the content of the chosen square, the number of possible second-order reactions (i.e. reactions involving two molecules) is determined. Randomly choose one of the eight nerest squares of the square chosen first for the same number of times as the number of possible second-order reactions. The rate constant is determined by the content of the neighboring squares chosen for each possible second-order reaction. The first-order reactions can happen regardless of the content of the neighboring squares and their rate is determined independent of the other squares.

3. Execute one of the possible reactions with a probability that is calculated as the reaction rate constant multiplied by a constant α. The value of α is chosen such that the maximum value of the sum of the probabilities of the possible reactions is less than 1.

Diffusion is viewed as a second-order reaction with rate constant D, and it is implemented by swapping the content of two squares. If complex molecules diffuse, swapping is done in such a way that the adjacency between the constituent molecules is maintained (see below). Diffusion and reaction across the grid boundaries are prohibited (no-flux boundary condition). The application of the above algorithm for N^2 times, where N^2 is the number of squares in the grid, is defined as one time-step of replicator dynamics. In the figures, the number of time-steps was always scaled so that the meaning of unit time has the same meaning as that of the ODE model that has the same rate constants as the CA model (precisely speaking, the number of time-steps was divided by α). The well-mixed condition (i.e. the case where $D = \infty$) can be simulated by slightly modifying the above algorithm. Namely, instead of choosing a second square from the neighborhood, the algorithm chooses it from the entire CA grid (i.e. global interactions). With this algorithm, the dynamics of the model is the same as that simulated by the Gillespie algorithm with the time being properly scaled.

The details of diffusion algorithm involving complex molecules are as follows. There are three possible cases in which swapping involves a complex molecule: (1) one molecule is forming a complex with some molecule, and the other is not; (2) the two molecules are forming a complex with each other; and (3) each molecule is forming a complex with other molecules. In case (1), let x and y be the squares chosen for diffusion (it does not matter which is chosen first), and let us suppose that x contains a non-complex molecule or is empty, and y contains a complex molecule. Let y' be the square containing the molecule with which the molecule in y is forming a complex. Then, swapping is done as follows: the molecule in x is moved to y'; the molecule in y is moved to x; the molecule originally in y' is moved to y. In case (2), the two molecules swap their position (i.e. the rotation of the complex molecule). In case (3), let us suppose that x also contains a complex, and let x' be the square containing the molecule which which the molecule in x is forming a complex. Then, swapping is done as follows: $x \to y'$; $x' \to y$; $y \to x'$; $y' \to x$, where arrows mean that the molecule originally in the left square will be moved to the right square.

In the algorithm described above, one actually has to take into account the fact that a complex molecule consist of two molecules. This fact means that the chance of its being chosen at the beginning of the algorithm described above is twice that of a single molecules. To take this effect into account, the rate constant of replication (κ), the complex dissociation ($1 - \text{Rrec}$ and $1 - \text{Drec}$) and the diffusion of complex molecules were halved when the probability of possible reactions were calculated. Furthermore, the above fact means that one of the eight neighbors of a molecule that constitutes a complex molecule is always another molecule that constitutes the same complex. To take this effect into account, if a complex molecule is chosen at the beginning of the algorithm, a neighboring square for the potential replication reaction is chosen from the seven nearest squares that excludes the square containing the molecule that forms the focal complex molecule. For the diffusion process, however, a neighboring square is chosen from the eight nearest squares since diffusion between two molecules forming the same complex molecule is treated as the rotation of the complex molecule.

As described in the main text, the change in the values of Rrec and Drec through mutations was obtained by adding a random number uniformly distributed in $[-\delta/2, \delta/2]$. Rrec and Drec were bounded in $[0, 1]$, where the boundaries were reflective so that mutations generate uniform distribution in the parameters in the absence of selection. To be precise, let x be the original value of a parameter and y be a random number drawn from $[-\delta/2, \delta/2]$. If $x + y < 0$, the parameter is set to $-x - y$. If $x + y > 1$, the parameter is set to $2 - x - y$.

The Compartment Model

Cellular Potts Model (CPM) is two-scale stochastic CA, in which the rules of updating the CA pertain to the scale of neighboring grid squares and to the scale of a “compartment” that consists of a number of grid squares [3, 4]. Each compartment has a unique state, which is assigned to the grid squares that constitute the compartment. There is also one state representing non-compartment state, called medium

state. The CA is updated with stochastic algorithm minimizing "energy" H defined as

$$H = \sum_{(i,j)\in S} J_{(i,j)} + \sum_k \lambda(v_k - V_k)^2, \tag{1}$$

where (i,j) denotes a pair of squares that are adjacent to each others, where adjacency is defined as the eight nearest squares (the Moore neighbors); S is a set of all such pairs (not permutations, but combinations); $J_{(i,j)}$ is energy associated with an interface between different compartments or between a compartment and the medium, and it depends on the state of square i and j as explained soon; k denotes a compartment, and it runs through all compartments in the summation; v_k denotes the volume of a compartment, i.e. the number of grid squares that constitute the compartment; V_k denotes the so-called target volume; λ is a parameter and is set to 1 throughout this study. The current study defines $J_{(i,j)}$ as follows: $J_{(i,j)} = 3$ if $i \neq j$, and i and j are compartment states (i.e. if the two squares belong to different compartments); $J_{(i,j)} = 1$ if $i \neq j$, and either i or j is the medium state; $J_{(i,j)} = 0$ if $i = j$. For these values of J, a compartment tends to be more often in contact with media than with other compartments. The algorithm of updating the CA runs as follows:

1. Chose one square (denoted by x_1) from the grid in a random order such that the same square is not chosen twice until every square is chosen.

2. Randomly chose one (denoted by x_2) of the four nearest squares of x_1 (von Neumann neighbors).

3. Calculate the energy difference (denoted by ΔH) by subtracting the current value of H from the value of H if the state of x_2 were copied to x_1.

4. If $\Delta H < 0$, copy the state of x_2 to x_1. If $\Delta H > 0$, copy the state of x_2 to x_1 with a probability $\exp(-\Delta H/T)$, where T is a parameter and is set to 1 throughout this study.

The application of this algorithm for N^2 times, where N^2 is the number of squares in the grid, is defined as one time-step of the CPM. The dynamics of the CPM is run for one time-step per every $\mathrm{Int}(M/\alpha)$ time-steps of the replicator dynamics, where $\mathrm{Int}(x)$ is the nearest integer to x; α is defined in the previous section; M was tuned to obtain a reasonable model behavior and was set to 6.62 throughout the current study. The division of compartments can happen once per CPM time-step for every compartment.

The simulation program of the models specified above was written in C++ programing language. The source code is available from the author upon request.

The ODE Models

The System Consisting of One Species of Rp and One Species of Dp

The following ordinary differential equations describe the population dynamics of a replicator system consisting of one species of Rp and one species of Dp under the assumption of infinite diffusion and population size. The concentration of $\mathrm{Rp^{RNA}}$, $\mathrm{Rp^{DNA}}$, $\mathrm{Dp^{RNA}}$ and $\mathrm{Dp^{DNA}}$ are denoted by Rp^R, Rp^D, Dp^R and Dp^D respectively. The concentration of complex molecules formed between catalysts and templates are denoted by C_x^y where x and y denote the catalyst and template respectively. The concentration of the resource ($\emptyset$) is denoted by θ. Rrec and Drec are denoted by ρ_x^R and ρ_x^D, respectively, where x denotes

which polymerase a ρ pertains to. The model was numerically solved by using GRIND [5].

$$
\begin{aligned}
\theta =& 1 - Rp^R - Rp^D - Dp^R - Dp^D - 2C^{Rp^R}_{Rp^R} - 2C^{Rp^D}_{Rp^R} - 2C^{Dp^R}_{Rp^R} - 2C^{Dp^D}_{Rp^R} \\
&- 2C^{Rp^R}_{Dp^R} - 2C^{Rp^D}_{Dp^R} - 2C^{Dp^R}_{Dp^R} - 2C^{Dp^D}_{Dp^R} \\
\frac{d}{dt}Rp^R =& - dRp^R \\
&- 2\rho^R_{Rp}Rp^R Rp^R + 2(1-\rho^R_{Rp})C^{Rp^R}_{Rp^R} + 3\kappa C^{Rp^R}_{Rp^R}\theta + 2dC^{Rp^R}_{Rp^R} \\
&- \rho^R_{Dp}Dp^R Rp^R + (1-\rho^R_{Dp})C^{Rp^R}_{Dp^R} + \kappa C^{Rp^R}_{Dp^R}\theta + dC^{Rp^R}_{Dp^R} \\
&- \rho^D_{Rp}Rp^R Rp^D + (1-\rho^D_{Rp})C^{Rp^D}_{Rp^R} + 2\kappa C^{Rp^D}_{Rp^R}\theta + dC^{Rp^D}_{Rp^R} \\
&- \rho^R_{Rp}Rp^R Dp^R + (1-\rho^R_{Rp})C^{Dp^R}_{Rp^R} + \kappa C^{Dp^R}_{Rp^R}\theta + dC^{Dp^R}_{Rp^R} \\
&- \rho^D_{Rp}Rp^R Dp^D + (1-\rho^D_{Rp})C^{Dp^D}_{Rp^R} + \kappa C^{Dp^D}_{Rp^R}\theta + dC^{Dp^D}_{Rp^R} \\
\frac{d}{dt}Rp^D =& - dRp^D + \kappa C^{Rp^R}_{Dp^R}\theta \\
&- \rho^D_{Rp}Rp^R Rp^D + (1-\rho^D_{Rp})C^{Rp^D}_{Rp^R} + \kappa C^{Rp^D}_{Rp^R}\theta + dC^{Rp^D}_{Rp^R} \\
&- \rho^D_{Dp}Dp^R Rp^D + (1-\rho^D_{Dp})C^{Rp^D}_{Dp^R} + 2\kappa C^{Rp^D}_{Dp^R}\theta + dC^{Rp^D}_{Dp^R} \\
\frac{d}{dt}Dp^R =& - dDp^R + \kappa C^{Dp^D}_{Rp^R}\theta \\
&- \rho^R_{Dp}Dp^R Rp^R + (1-\rho^R_{Dp})C^{Rp^R}_{Dp^R} + \kappa C^{Rp^R}_{Dp^R}\theta + dC^{Rp^R}_{Dp^R} \\
&- \rho^D_{Dp}Dp^R Rp^D + (1-\rho^D_{Dp})C^{Rp^D}_{Dp^R} + \kappa C^{Rp^D}_{Dp^R}\theta + dC^{Rp^D}_{Dp^R} \\
&- \rho^R_{Rp}Rp^R Dp^R + (1-\rho^R_{Rp})C^{Dp^R}_{Rp^R} + 2\kappa C^{Dp^R}_{Rp^R}\theta + dC^{Dp^R}_{Rp^R} \\
&- 2\rho^R_{Dp}Dp^R Dp^R + 2(1-\rho^R_{Dp})C^{Dp^R}_{Dp^R} + 2\kappa C^{Dp^R}_{Dp^R}\theta + 2dC^{Dp^R}_{Dp^R} \\
&- \rho^D_{Dp}Dp^R Dp^D + (1-\rho^D_{Dp})C^{Dp^D}_{Dp^R} + \kappa C^{Dp^D}_{Dp^R}\theta + dC^{Dp^D}_{Dp^R} \\
\frac{d}{dt}Dp^D =& - dDp^D + \kappa C^{Dp^R}_{Dp^R}\theta \\
&- \rho^D_{Rp}Rp^R Dp^D + (1-\rho^D_{Rp})C^{Dp^D}_{Rp^R} + \kappa C^{Dp^D}_{Rp^R}\theta + dC^{Dp^D}_{Rp^R} \\
&- \rho^D_{Dp}Dp^R Dp^D + (1-\rho^D_{Dp})C^{Dp^D}_{Dp^R} + 2\kappa C^{Dp^D}_{Dp^R}\theta + dC^{Dp^D}_{Dp^R}
\end{aligned}
$$

$$\frac{d}{dt}C^{Rp^R}_{Rp^R} = \rho^R_{Rp} Rp^R Rp^R - (1-\rho^R_{Rp})C^{Rp^R}_{Rp^R} - \kappa C^{Rp^R}_{Rp^R}\theta - 2dC^{Rp^R}_{Rp^R}$$
$$\frac{d}{dt}C^{Rp^D}_{Rp^R} = \rho^D_{Rp} Rp^R Rp^D - (1-\rho^D_{Rp})C^{Rp^D}_{Rp^R} - \kappa C^{Rp^D}_{Rp^R}\theta - 2dC^{Rp^D}_{Rp^R}$$
$$\frac{d}{dt}C^{Dp^R}_{Rp^R} = \rho^R_{Rp} Rp^R Dp^R - (1-\rho^R_{Rp})C^{Dp^R}_{Rp^R} - \kappa C^{Dp^R}_{Rp^R}\theta - 2dC^{Dp^R}_{Rp^R}$$
$$\frac{d}{dt}C^{Dp^D}_{Rp^R} = \rho^D_{Rp} Rp^R Dp^D - (1-\rho^D_{Rp})C^{Dp^D}_{Rp^R} - \kappa C^{Dp^D}_{Rp^R}\theta - 2dC^{Dp^D}_{Rp^R}$$
$$\frac{d}{dt}C^{Rp^R}_{Dp^R} = \rho^R_{Dp} Dp^R Rp^R - (1-\rho^R_{Dp})C^{Rp^R}_{Dp^R} - \kappa C^{Rp^R}_{Dp^R}\theta - 2dC^{Rp^R}_{Dp^R}$$
$$\frac{d}{dt}C^{Rp^D}_{Dp^R} = \rho^D_{Dp} Dp^R Rp^D - (1-\rho^D_{Dp})C^{Rp^D}_{Dp^R} - \kappa C^{Rp^D}_{Dp^R}\theta - 2dC^{Rp^D}_{Dp^R}$$
$$\frac{d}{dt}C^{Dp^R}_{Dp^R} = \rho^R_{Dp} Dp^R Dp^R - (1-\rho^R_{Dp})C^{Dp^R}_{Dp^R} - \kappa C^{Dp^R}_{Dp^R}\theta - 2dC^{Dp^R}_{Dp^R}$$
$$\frac{d}{dt}C^{Dp^D}_{Dp^R} = \rho^D_{Dp} Dp^R Dp^D - (1-\rho^D_{Dp})C^{Dp^D}_{Dp^R} - \kappa C^{Dp^D}_{Dp^R}\theta - 2dC^{Dp^D}_{Dp^R}$$

The System Consisting of Two Species of Rp and One Species of Dp

The ODE model described in the previous section was extended to include one extra species of Rp (Rp' in the main text). The equations were obtained from a computer program written by the author, and they are omitted from inclusion for the sake of space (the model consists of 24 equations and contains the above equations as a subset).

The Models that Take Account of the Trade-off between Replication and Transcription

To examine the effect of the trade-off between replication and transcription on the evolution of DNA, we slightly modified the original models such that each molecule was assigned two additional parameters: RecRp and RecDp for recognition by Rp and recognition by Dp, respectively. These parameters allow a template to control how well it is recognized by a polymerase. More precisely, if, say, the template is RNA and the catalyst is Rp, the rate constant of complex formation between the template and the catalyst was $\mathrm{Rrec} \times \mathrm{RecRp}$, whereas the rate constant of complex dissociation was $1 - \mathrm{Rrec} \times \mathrm{RecRp}$. Simulations with the modified models showed essentially the same results as those with the original model. The simulations also showed that, in the surface model, the average value of RecRp was slightly smaller than the value of RecDp for both transcriptase and DNA replicase ($\mathrm{RecRp} \approx 0.8$ to $\mathrm{RecDp} \approx 1$), but not for RNA replicase. In the compartment model, this was the case only for the dual specificity Rp. This result is in agreement with the expectation that the trade-off between replication and transcription causes a selection pressure on templates to reduce the time they spend being transcribed and with the results (described in the main text) that the genetic information is transmitted through DNA replication for transcriptase and DNA replicase in the surface model and dual specificity Rp in the compartment model. These results indicate that, although the effect of the trade-off between replication and transcription is not negligible as the models exhibited a reduction in the transcription activity, it is not large enough to qualitatively change the main results obtained with the original models. This robustness of the results against the effect of the trade-off between replication and transcription can be rationalized in terms of a well-known fact from the group selection theory as described in the main text.

It has to be, however, added that the current examination of the trade-off between replication and transcription is incomplete for several reasons. First, the way in which RecRp and RecDp determined the rate constant of complex formation was chosen arbitrarily: there is a number of ways in which these parameters can be combined. Furthermore, if the discrimination of catalysts by templates were to be taken into account, it would be desirable, for the sake of symmetry, to also take into account the discrimination of templates by catalysts (beyond the discrimination between RNA and DNA templates). However, more importantly, the discrimination of catalysts by templates should not be determined solely by the property of templates alone as was the case in the modified model described above, but should be determined by the interactions between templates and catalysts. Therefore, in order to fully take into account the evolution of the interactions between templates and catalysts—and, hence, the trade-off between replication and transcription—there is clearly a severe limitation in the current model formalism, where individual molecules are characterized by a small number of numerical parameters and the interactions between them are determined through the combination of these parameters. However, to go beyond the original model formalism is beyond the scope of the present study (cf. [6]).

Nevertheless, it is an assuring fact that the modification to the original models described above, which is the minimal extension to take into account the trade-off between replication and transcription, did not qualitatively change the results described in the main text.

References

1. Takeuchi N, Hogeweg P (2009) Multilevel selection in models of prebiotic evolution II: A direct comparison of compartmentalization and spatial self-organization. PLoS Comput Biol 5: e1000542.

2. Takeuchi N, Hogeweg P (2007) The role of complex formation and deleterious mutations for the stability of RNA-like replicator systems. J Mol Evol 65: 668-686.

3. Graner F, Glazier JA (1992) Simulation of biological cell sorting using a two-dimensional extended potts model. Phys Rev Lett 69: 2013-2016.

4. Anderson ARA, Chaplain MAJ, Rejniak KA, editors (2007) Single-Cell-Based Models in Biology and Medicine, Basel: Birkhäuser Verlag, chapter II. pp. 77-167.

5. Boer RJ de, Pagie L (2005). GRIND. URL `http://theory.bio.uu.nl/rdb/software.html`.

6. Takeuchi N, Hogeweg P (2008) Evolution of complexity in RNA-like replicator systems. Biol Direct 3: 11.
